# Growth and Wood Trait Relationships of *Alnus glutinosa* in Peatland Forest Stands With Contrasting Water Regimes

**DOI:** 10.3389/fpls.2021.788106

**Published:** 2022-01-12

**Authors:** Alba Anadon-Rosell, Tobias Scharnweber, Georg von Arx, Richard L. Peters, Marko Smiljanić, Simon Weddell, Martin Wilmking

**Affiliations:** ^1^Institute of Botany and Landscape Ecology, University of Greifswald, Greifswald, Germany; ^2^CREAF, Edifici C, Cerdanyola del Vallès, Catalonia, Spain; ^3^Swiss Federal Institute for Forest, Snow and Landscape Research WSL, Birmensdorf, Switzerland; ^4^Oeschger Centre for Climate Change Research, University of Bern, Bern, Switzerland; ^5^Forest Is Life, TERRA Teaching and Research Centre, Gembloux Agro Bio-Tech, University of Liège, Liège, Belgium

**Keywords:** alder carr, hydraulic traits, peatland restoration, waterlogging, wet forest, wood density, xylem anatomy

## Abstract

Human-driven peatland drainage has occurred in Europe for centuries, causing habitat degradation and leading to the emission of greenhouse gases. As such, in the last decades, there has been an increase in policies aiming at restoring these habitats through rewetting. Alder (*Alnus glutinosa* L.) is a widespread species in temperate forest peatlands with a seemingly high waterlogging tolerance. Yet, little is known about its specific response in growth and wood traits relevant for tree functioning when dealing with changing water table levels. In this study, we investigated the effects of rewetting and extreme flooding on alder growth and wood traits in a peatland forest in northern Germany. We took increment cores from several trees at a drained and a rewetted stand and analyzed changes in ring width, wood density, and xylem anatomical traits related to the hydraulic functioning, growth, and mechanical support for the period 1994–2018. This period included both the rewetting action and an extreme flooding event. We additionally used climate-growth and climate-density correlations to identify the stand-specific responses to climatic conditions. Our results showed that alder growth declined after an extreme flooding in the rewetted stand, whereas the opposite occurred in the drained stand. These changes were accompanied by changes in wood traits related to growth (i.e., number of vessels), but not in wood density and hydraulic-related traits. We found poor climate-growth and climate-density correlations, indicating that water table fluctuations have a stronger effect than climate on alder growth. Our results show detrimental effects on the growth of sudden water table changes leading to permanent waterlogging, but little implications for its wood density and hydraulic architecture. Rewetting actions should thus account for the loss of carbon allocation into wood and ensure suitable conditions for alder growth in temperate peatland forests.

## Introduction

Forest peatlands are widely distributed around the globe and represent 20–25% of global peatland cover (Zoltai and Martikainen, 1996). These ecosystems accumulate large amounts of carbon (C) as peat, but for centuries have been drained for agriculture, forestry, and peat extraction (Joosten, 2009). Human-driven peatland drainage has led to a drastic reduction in the occurrence of peatland forests, resulting in large negative impacts on biodiversity and converting them into C sources with a consequent positive feedback on climate change (Joosten et al., 2016). In the last decades, rewetting policies have been introduced with the aim to restore drained peatlands and their ecosystem functions (Cris et al., 2014; Tanneberger et al., 2021) but the extent of their long-term success is still largely unknown. In peatland forests, this success heavily depends on the acclimation capacity of the tree species to increased water tables.

Black alder [*Alnus glutinosa* (L.) Gaernt, hereafter, alder] is a tree species naturally widespread in the wet areas across Europe, making up 1% of forest cover in most of the countries (but *c*. 5% in North Central Europe, Claessens et al., 2010). Specifically, it grows in wet areas, such as riverbanks, wetlands, and lake shores, but due to historical peatland drainage, the occurrence of alder wet forests has been reduced (Prieditis, 1997). Alder is a water-demanding species with poor stomatal regulation, and its rooting system is well-adapted to soil saturation, with surface and deep root branching. It is a pioneer species that can fix atmospheric nitrogen, thanks to its symbiosis with the bacterium, *Frankia alni* (McVean, 1956; Claessens et al., 2010); it is a peat forming species (Barthelmes, 2009), and it is of economic value for timber production (Claessens et al., 2010). Even though it is a waterlogging-tolerant species (Glenz et al., 2006; Niinemets and Valladares, 2006), several studies have reported the negative effects of prolonged flooding and water level fluctuations on alder radial growth (Laganis et al., 2008; Douda et al., 2009; Rodríguez-González et al., 2010; Tulik et al., 2020) and high water table levels are known to decrease the wood quality of alder (Barthelmes, 2009). Previous attempts at rewetting drained alder forests that led to sudden changes in the water table and permanent waterlogging caused severe tree damage and ultimately trees and forests die-off (Bönsel, 2006). Understanding how alder growth, functioning, and subsequent survival may be affected by rewetting actions is paramount to design effective protocols that ultimately restore the ecosystem functions of alder wet forests and preserve their wood production potential as much as possible.

The physiological and wood structural modifications of trees are essential for facing environmental changes. Anatomical structures may be negatively affected by waterlogging even in flood-tolerant species due to a reduction of cambial activity (Kozlowski, 1984). Indeed, vessel size has been shown to respond negatively to high water table levels in alder after flooding (Tulik et al., 2020). Studies on other flood-tolerant species, such as *Quercus robur* L. have also reported a decrease in the vessel size in response to flooding (Tumajer and Treml, 2016), as well as the appearance of “flood rings” after spring flooding, characterized by having especially narrow earlywood vessels caused by the cessation of earlywood vessel development (Copini et al., 2016). Conversely, evidence of a positive relationship between the duration of flooding and both vessel size and number were found in ash trees (*Fraxinus excelsior* L.) in a floodplain in Poland (Koprowski et al., 2018). Such changes in the structural characteristics of the woody hydraulic architecture affect water transport efficiency and safety (Hacke and Sperry, 2001), especially when facing changes in the water regime. This might be especially important for species with less sensitive stomatal regulation (and thus low capacity of regulating transpiration), such as alder (Herbst et al., 1999), which may be expected to particularly rely on wood anatomical adjustments at the branch, the stem, and the root level. Wood anatomical adjustments may thus provide the basis to preserve tree growth and functioning in this species when facing water table changes.

The adjustment of the wood anatomical architecture to environmental fluctuations may be linked to other structural modifications in the xylem fiber cells and, consequently, the wood density (Ziemińska et al., 2013; Björklund et al., 2021). Wood density is an important wood property for timber production and wood utilization, and it is strongly related to tree C sequestration (Babst et al., 2014). Even though waterlogging seems to strongly affect the biosynthesis of cell wall components (Kreuzwieser and Rennenberg, 2014, and references therein), a previous study has shown no clear response of alder wood density to water table changes (van der Maaten et al., 2015). Regardless, tissue-scale trade-offs (e.g., vessel vs. fiber tissue, lumen area vs. cell wall thickness) and trait relationships in the alder xylem are unknown (but see Kiaei et al., 2016), and the adjustments of growth and the wood anatomical structure after rewetting, with their implications for wood density, are poorly understood.

In this study, we analyzed the growth, wood density, and xylem anatomy of alder in a drained and in a rewetted stand. Our aim was to assess the effects of changing water regimes (rewetting and extreme flooding) on alder growth and wood characteristics at these two stands, representative of common alder-dominated communities in central Europe. We specifically had the following questions: (i) What are the growth, wood density, and xylem anatomical patterns in alder stands with contrasting water regimes? (ii) How does alder growth and wood traits respond to rewetting and extreme flooding? (iii) How is wood density linked to structure-function properties of the wood in alder?

## Materials and Methods

### Study Sites

**FIGURE 1:**
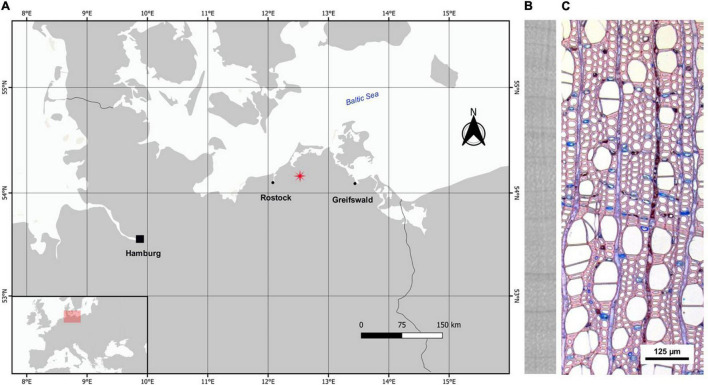
**(A)** Study area in NE Germany, **(B)** x-radiography of alder wood, **(C)** thin section of alder stem for xylem anatomical analysis. Vessels, fibers, ray and axial parenchyma, and a ring boundary (in the middle) are visible in the section, which belongs to tree ADAG01 from the drained stand.

The study area is located in Wöpkendorf, NE Germany (Figure 1A). We selected a long-term drained (54° 08′ 06″ N, 12° 32′ 11″ E, 44 m a.s.l., *c.* 0.27 ha) and a recently rewetted stand (54° 07′ 37″ N, 12° 29′ 04″ E, 37 m a.s.l., *c.* 0.75 ha) within a deciduous fen woodland dominated by alder. Both stands were drained for wood pasture at the end of the eighteenth century. A rewetting action took place in 2003 in the rewetted stand, when the ditches were filled to stop the drainage (Bönsel, 2006). The drained alder stand is a mixed stand of alder and a few ash individuals (*Fraxinus excelsior* L.). The understory is dominated by *Urtica dioica* L. and *Rubus idaeus* L. (*c.* 45 and 40% cover, respectively) and the peat layer is rather thin (*c.* 60 cm) and strongly degraded. The rewetted stand is a monospecific alder stand and its understory is dominated by *Carex riparia* Curtis (*c.* 70% cover) with *Glyceria fluitans* (L.) R. Br. and *Solanum dulcamara* L. (*c.* 30 and 20% cover, respectively). The peat layer in the rewetted stand is thicker and reaches over 2 m. Both stands are representative of peatland forest types present in the region and are study sites within the interdisciplinary joint project, WETSCAPES that aims to develop scientific principles for sustainable cultivation of wet peatlands, especially focusing on drained peatlands that have been rewetted (Jurasinski et al., 2020).

The mean annual temperature in the study area was 8.8°C and the mean annual precipitation was 601 mm (period 1981–2010). January is the coldest month (mean temperature of 0.7°C, mean precipitation of 46 mm), and July is the warmest (mean temperature of 17.8°C, mean precipitation of 59 mm; Germany’s National Meteorological Service; Supplementary Figure 1). In 2011, the study area received extremely high precipitation during summer, with an increase of July precipitation of about 340% relative to the climate normal of 1961–2010 (Supplementary Figure 1), causing flooding in the area.

### Tree Sampling and Sample Preparation

Between spring 2018 and spring 2019, we sampled 16 trees in the drained stand and 22 trees in the rewetted stand. All trees were upright, single-stemmed, and of similar size. We took two increment cores per tree with a 4.3 mm increment borer (Haglöf, Sweden); we labeled each tree and recorded tree height and diameter at breast height (DBH; Supplementary Table 1 and Supplementary Figure 2). We additionally recorded tree height and DBH for all surrounding trees at both stands (Supplementary Figure 2).

Since the standard optical measurements of tree-ring widths (TRW) in alder can be difficult to take because of unclear ring borders and color variations, we measured TRW on radiographic images taken with an Itrax Multiscanner (Cox Analytical Systems, Sweden). We mounted one of the two cores per tree on wooden holders and cut 1.2 mm thick laths parallel to the transverse wood surface with a twin-blade saw (Walesch Electronic, Switzerland). We obtained grayscale radiographic images with a spatial resolution of 20 μm (Figure 1B).

For the quantitative xylem anatomical analysis, we used the second core in a subset of five trees per stand (ten trees in total). We first cut each core in several 3–4 cm pieces and cut a 15 μm-thick section of each piece with a rotary microtome (Leica RM 2245, Leica Microsystems GmbH, Wetzlar, Germany). We stained the sections with a 1:1 mixture of safranin and astra blue, rinsed them with ethanol solutions of increasing concentration (50, 70, and 96%) and embedded them in Euparal. Finally, we dried them on a drying bench at 65–70°C for 48 h. We photographed the sections at 100 times magnification with a digital camera mounted on a microscope Leica DM 2500 (Leica Microsystems GmbH, Wetzlar Germany). We took several images per section with 30% overlap and stitched them with the open-source software ImageJ (Abràmoff et al., 2004; Preibisch et al., 2009; Schindelin et al., 2012) to obtain high-resolution images of each section (Figure 1C).

### Tree-Ring Widths, Wood Density, and Anatomical Measurements

We measured TRW on the radiographic images with CooRecorder v. 9.3.1 and visually and statistically crossdated the TRW series with CDendro (Cybis Elektronik and Data AB, Sweden). For each ring on the same image, we also measured the average ring density (AVD) with CooRecorder, which is the AVD over the whole ring, and the maximum latewood density (MXD), which is the highest density value of the latewood taken from the 20% darkest pixels within the ring (Björklund et al., 2019).

We used the software ROXAS v3.0 (von Arx and Carrer, 2014) to quantify xylem anatomical parameters and ring area and width in the anatomy-selected trees. The borders of the tree rings were drawn manually based on the cross-dating procedure previously performed for the same trees. For each annual ring, we quantified xylem anatomical parameters related to hydraulic efficiency, spatial vessel distribution and connectivity, wood density, and growth. Thus, we measured hydraulically weighted mean vessel diameter (Dh, calculated as Σ*d*5/Σ*d*4, where *d* is the cross-sectional mean lumen diameter of each vessel; Kolb and Sperry, 1999), theoretical hydraulic conductivity (Kh) based on Hagen-Poiseuille’s law (Tyree and Zimmerman, 2002) and theoretical xylem-specific hydraulic conductivity (Ks) calculated as Kh/ring area as parameters related to hydraulic efficiency. We studied spatial vessel distribution and connectivity with the parameters vessel density (CD, number of vessels per area) and vessel grouping index (RVGI, mean number of vessels per group; von Arx et al., 2013). We measured fiber cell wall thickness (CWT) in all fibers within the ring area analyzed as a parameter related to wood density and mechanical support (Ziemińska et al., 2013; Prendin et al., 2017). Finally, we measured the number of vessels per tree ring (CNo), mean TRW, and ring area (RA) as growth-related parameters. For all these parameters, we obtained a mean value for each annual ring.

We used anatomical data to calculate wood density profiles. For this, we divided each tree ring in the image into 50 μm-wide sectors (*s*) parallel to the ring border (corrected for the ring boundary curvature, Supplementary Figure 3). The size of the sectors was chosen so that they could contain at least one row of complete vessels. We calculated the mean density for each sector assuming a fixed density of wall material (*y* = 1.504 g cm-3; Kellogg and Wangaard, 1969) using the following equation (modified from Peters et al., 2020):


(1) $$\rho_{s}=\gamma \left(\sum_{i=0}^{c}\left[\frac{\pi \left(\alpha_{s}+\bar{CWT_{s}}\right)\left(\beta_{s}+\bar{CWT_{s}}\right)-A_{s}}{\pi \left(\alpha_{s}+\bar{CWT_{s}}\right)\left(\beta_{s}+\bar{CWT_{s}}\right)}\right]\right)$$

where the wall area of each cell (*c*, both for vessels and fibers) was calculated using CWT and cell lumen area (*A*, assuming an elliptical shape using α and β). We excluded the area of ray parenchyma from the equation, because our samples only showed scattered uniseriate rays and, within the 4.3 mm-wide sections, these were not representative. We excluded the area of axial parenchyma because it was also poorly representative within the sections analyzed. We built wood anatomical density profiles for all types of cells combined (wood anatomical density), and separately for the vessel tissue and the fiber tissue. See Peters et al. (2020) for more details on the method used.

### Statistical Analysis

We calculated the common chronology statistics to assess the quality of the chronology for each stand, including mean inter-series correlation (Rbar), expressed population signal (EPS) and Gleichlaeufigkeit (GLK; Bunn, 2008).

To reduce the amount of xylem anatomical parameters for the analyses, we first explored the relationship between these parameters with a principal component analysis (PCA). Then, to evaluate the differences in alder growth, x-ray density (both at stand level and for the anatomy-selected trees only) and the selected xylem anatomical parameters between stands and between study periods (before and after rewetting, before and after the extreme flooding), we used linear mixed-effects models fitted with the restricted maximum likelihood (REML) estimation method. Models included stand, period (pre-rewetting 1994–2002; post-rewetting 2003–2010; post-flooding 2011–2017) and their interactions as fixed effects. For stand-level growth analysis, year 2018 was also included, as there was enough data for statistical analyses. For AVD and MXD, analyses only included up to year 2016, as the ring closest to the bark (for some trees 2017, for others 2018) showed extremely high values due to edge effects in the X-ray procedure. Models included cumulative DBH (cDBH) when it was significant to account for allometric effects on response variables. The random effects structure included tree identity as a random intercept. We applied a residual auto-correlation structure (corAR1, Pinheiro et al., 2019) to account for the temporal dependence of the residuals in measurements from the same tree. We graphically evaluated the assumptions of normality and homoscedasticity of residuals (Zuur et al., 2010), applying log-transformations, when necessary, to reach these assumptions. We tested for significance with ANOVA tests and performed *post-hoc* tests to test for significant differences between factor levels. For those parameters in which a change after 2011 was evident but not represented by the models (which were using period as a whole and not annual changes), we calculated the annual relative change after the extreme event. For this, we calculated the mean of the 6 years prior to the event as a reference for each stand separately and calculated the deviation (percent change) from the respective reference. We used Welch’s *t*-tests, suitable when variances are unequal and data unbalanced, to test for significant differences each year after the event with respect to the reference (Welch, 1947). We also used linear mixed-effects models to evaluate differences in the anatomical density components (i.e., wood anatomical density, fiber anatomical density, and vessel anatomical density) between stands and periods. Finally, we used linear and nonlinear regressions to analyze relationships between anatomical traits.

To determine the climate-growth and climate-density relationships, we detrended TRW series of individual trees to remove long-term trends (i.e., age/size-related signals) and retain high-frequency variability by fitting a 30-year cubic smoothing spline (Cook and Kairiukstis, 1990). We followed the same procedure for AVD and MXD. We calculated bootstrapped Pearson’s correlation coefficients based on 1,000 iterations between each stand chronology and climate variables (i.e., monthly mean temperature and monthly precipitation sums) starting from the previous June to September of the current year over the period 1950–2018 (for density parameters only until 2016). We also tested for climate-growth relationships for winter (previous December to February), spring (March to May), summer (June to August), and autumn (September to November) seasons over the same period. To explore lagged responses to precipitation, we tested for growth and density correlations with precipitation sums one and 2 years prior to the growing year, respectively. We obtained climate data for our study stands from the German Weather Service (DWD). We used 1 km^2^ grid resolution, which interpolates data from station data (Müller-Westermeier, 1995). We extracted the monthly data for mean temperature and precipitation of the grid cells where our forest stands were located.

We used R v4.0.3 for all statistical analyses (R Core Team, 2020). We used the package “dplR” to process tree-ring chronologies and detrend data (Bunn, 2008) and the package “treeclim” (Zang and Biondi, 2015) for climate-growth and climate-density analyses. We used the “nlme” package (Pinheiro et al., 2019) for linear mixed-effects models, the “emmeans” package (Lenth, 2020) for *post-hoc* tests and “ggplot2” for graphical display (Wickham, 2016).

## Results

### Tree Growth and Wood Density at Stand Level

**FIGURE 2:**
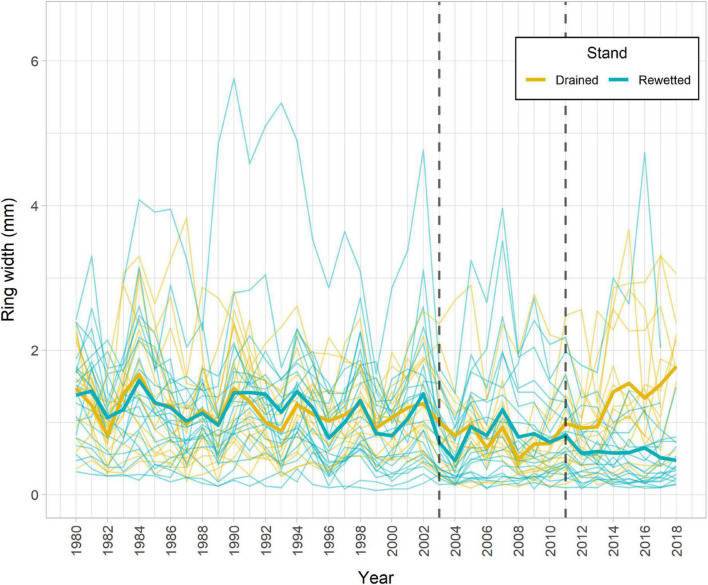
Alder tree-ring width (TRW) chronology for the study period 1994–2018 for both stands, including some years prior to 1994. Individual tree chronologies are shown in thin lines and mean average chronologies for each stand are shown in thick lines. Vertical dotted lines indicate the year of rewetting (2003) and the year of extreme flooding (2011).

The main descriptive TRW chronology statistics for the drained and the rewetted stand, respectively, were as follows: Rbar = 0.21, 0.33; EPS = 0.81, 0.92; and GLK = 0.61, 0.68. The TRW chronologies revealed three periods in which alder growth differed between the sites, before 1944, between 1952 and 1968, and after 2011 (Supplementary Figure 4). We focused our analyses on the period between 1994 and 2018, which encompasses the period running from 10 years before the last rewetting action in the area until present, including the extreme flooding event in 2011 (Figure 2). We found a significant effect of Period and Stand × Period on TRW (Table 1). *Post-hoc* tests showed a significant divergence between stands after 2011 (drained > rewetted, *P* = 0.001) and a trend to reduce TRW from 1994 toward 2018 in the rewetted stand (period before 2003 > period 2003–2010, *P* < 0.001; period before 2003 > period post-2011, *P* < 0.001). In the drained stand, TRW decreased after 2003 (period before 2003 > period 2003–2011, *P* < 0.001) but increased again after 2011 (period post-2011 > period 2003–2010, *P* < 0.0001). Welch’s *t*-tests on the relative change in TRW after the 2011 event showed that TRW of alder in the drained stand significantly increased 92% in the third year after the event with respect to the reference (*P* = 0.012) and continued to do so until 2018, when values were 140% higher than the reference (*P* = 0.005). In the rewetted stand, TRW decreased 35% the year after the event (*P* = 0.039), a value that was maintained over the period 2012–2018 (*P* < 0.05 except for year 2016, n.s. Supplementary Figure 5).

**TABLE 1:** Results of the ANOVA tests for each variable at (a) stand level and for the (b) anatomy-selected trees.

Variable	cDBH			Stand			Period			Stand × Period		
	df	*F*	*P*	df	*F*	*P*	df	*F*	*P*	df	*F*	*P*
**a) Stand level**												
TRW	–	–	–	1,36	3.67	**0.063**	2,857	35.16	**<0.001**	2,857	11.73	**<0.001**
AVD	1,772	3.24	**0.072**	1,35	0.45	0.505	2,772	5.66	**0.004**	2,277	2.96	**0.052**
MXD	1,768	5.62	**0.018**	1,35	1.11	0.299	2,768	4.89	**0.008**	2,768	2.83	**0.060**
**b) Anatomy-selected trees**												
TRW	–	–	–	1,8	0.35	0.568	2,220	3.61	**0.029**	2,220	0.53	0.590
CD	–	–	–	1,8	0.59	0.465	2,219	3.00	**0.052**	2,219	0.98	0.379
Dh	–	–	–	1,8	0.25	0.628	2,220	0.30	0.742	2,220	4.66	**0.010**
Ks	–	–	–	1,8	0.47	0.514	2,219	0.96	0.383	2,219	3.68	**0.027**
CWT	1,197	5.45	**0.021**	1,7	1.73	0.23	2,197	0.37	0.689	2,197	0.35	0.704

*Cumulative diameter at breast height (cDBH) was only kept in the models when it was significant or marginally significant (P < 0.10). Degrees or freedom (df), F-statistics (F), and P-values (P) are shown. TRW, tree-ring width; AVD, average ring density; MXD, maximum latewood density; CD, vessel density; Dh, hydraulically weighted mean vessel diameter; Ks, theoretical xylem-specific hydraulic conductivity; CWT, cell wall thickness. Significant (P < 0.05) and marginally significant (0.10 > P ≥ 0.05) P-values are in bold face.*

Average ring density and MXD chronologies of alder were influenced by cDBH and showed a significant Period effect with smaller values between 2003 and 2010 than before 2003 at both stands (*post-hoc* test *P* = 0.019 and 0.008, respectively), and a marginally significant Stand × Period effect. For AVD, *post-hoc* tests showed that in the rewetted stand, values were larger before 2003 than in the other two periods (*P* < 0.001 and 0.014, respectively). For MXD, *post-hoc* tests showed marginally significant higher values before 2003 than between 2003 and 2010 in the drained stand (*P* = 0.066), and larger values before 2003 than in the other two periods in the rewetted stand (*P* = 0.072 and 0.005, respectively; Table 1; Supplementary Figure 6).

### Climate-Growth and Climate-Density Relationships

Overall, we found mild correlations between growth and climate variables, and between density and climate variables (Supplementary Figures 7, 8). The highest correlation coefficients corresponded to previous October temperature and growth at the drained stand, and previous November temperature and AVD at the rewetted stand, but significant correlations with precipitation were more common than with temperature for these two parameters. MXD poorly correlated with climate variables (Supplementary Figure 7). When grouping climate variables in seasonal variables (Supplementary Figure 8), we found a positive correlation between growth and winter precipitation (from December of the previous year to February) at both stands. AVD only correlated significantly with winter precipitation in the rewetted stand, MXD only correlated negatively with autumn temperature also in the rewetted stand. When exploring lagged responses to precipitation, we found significant correlations only in the rewetted stand. Growth correlated with the annual precipitation sum of 2-years-prior to the growing year (*r* = −0.28). For AVD, we found a positive correlation with annual precipitation of the year prior to the growing year (*r* = 0.24). For MXD, we found a negative correlation with the annual precipitation of the year prior to the growing year (*r* = −0.23).

### Growth and Xylem Anatomy in the Anatomy-Selected Trees

Based on the PCA analysis, we highlight the results of statistical analysis for a sub-selection of traits covering water transport and mechanical support functions, namely TRW, CD, Dh, Ks, and CWT (Supplementary Figure 9). Information on other parameters of interest (i.e., CNo, Kh, and RVGI) can be found in Supplementary Table 2 and in Supplementary Figures 10, 11.

**FIGURE 3:**
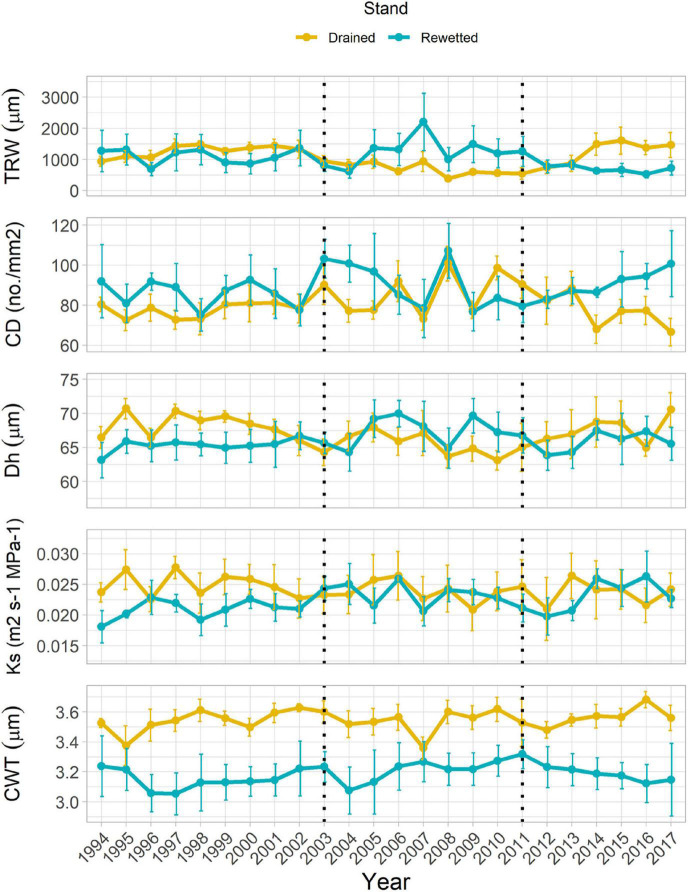
Tree-ring width (TRW), vessel density (number of vessels per area, CD), hydraulically weighted mean vessel diameter (Dh), xylem-specific hydraulic conductivity (Ks) and cell wall thickness (CWT) of fibers in the study stands (means ± SE). Vertical dotted lines indicate the year of rewetting (2003) and the year of extreme flooding (2011).

**FIGURE 4:**
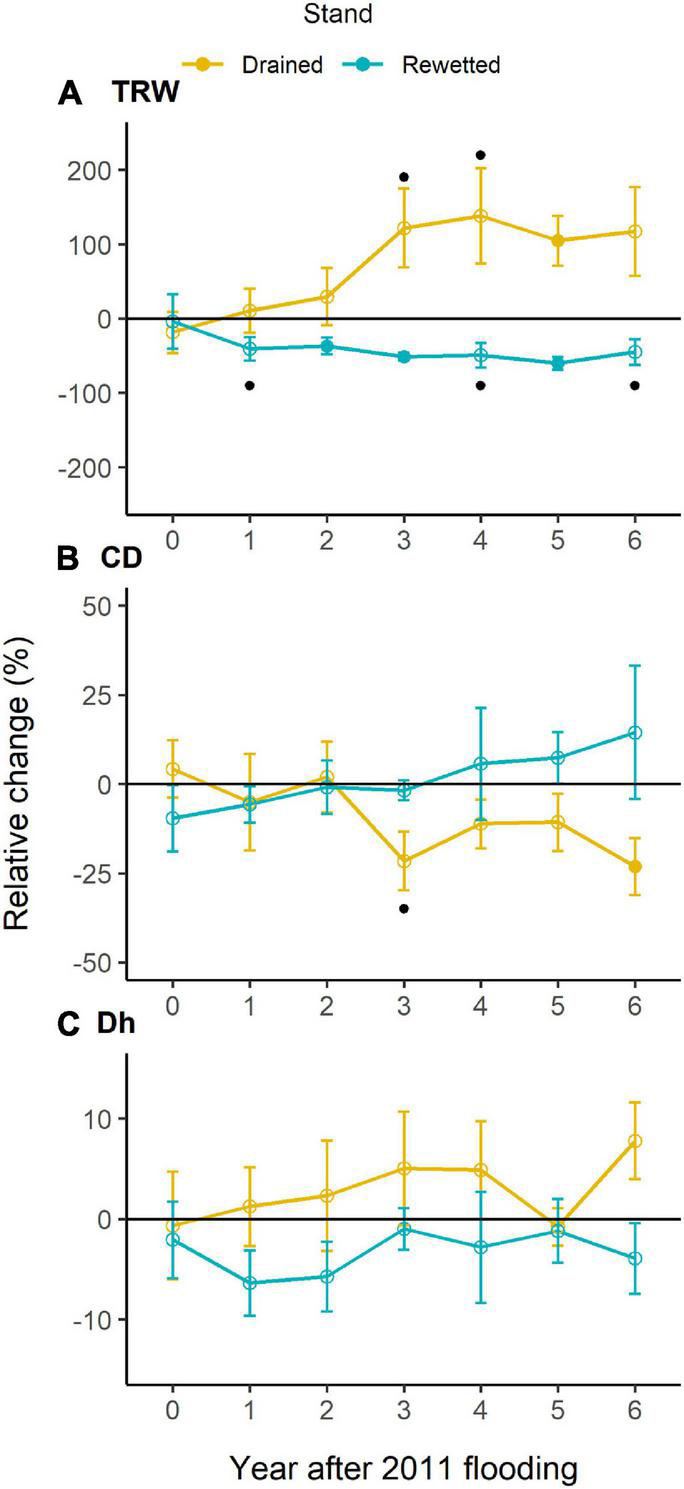
Relative change (means ± SE) of **(A)** tree-ring width (TRW), **(B)** vessel density (CD), and **(C)** hydraulically weighted mean vessel diameter (Dh) after 2011 flooding with respect to the reference (mean of 6 years prior to 2011 for each stand) in both stands. Year 0 corresponds to 2011. Empty circles represent non-significant change values (*P* ≥ 0.10), filled circles represent significant changes with respect to the reference (*P* < 0.05). Dots indicate marginally significant changes (0.05 ≤ *P* < 0.10).

Tree-ring width in the anatomy-selected trees ranged between 0.07 and 5.15 mm from 1994 until 2017. It showed a significant Period effect, with significantly larger values in the period before 2003 (1.19 ± 2.13 mm, *post-hoc* test *P* = 0.035) and marginally significant larger values for the period 2011–2017 (0.93 ± 1.74 mm, *post-hoc* test *P* = 0.071) than for the period 2003–2010 (0.95 ± 2.21 mm; Table 1 and Figure 3). We did not find a Stand × Period effect with the linear mixed-effects model, but Welch’s tests showed a significant TRW increase in the drained and a significant decrease in the rewetted stand after the extreme flooding in 2011. In the drained stand, TRW decreased in the year of the extreme event, and the increase started 1 year after, although a significant change only occurred in 2016 (122% increase with respect to the reference, *P* = 0.036). In the rewetted stand, TRW decreased 41% already 1 year after the event (*P* = 0.061, marginally significant) and was maintained until the end of the study (Figure 4A). CD showed a marginally significant Period effect (Table 1; period 2003–2010 > period 1994–2002). We did not find a Stand × Period effect with the linear mixed-effects model, but Welch’s tests showed a 22% decrease (*P* = 0.057, marginally significant) 3 years after the event in the drained stand, which occurred again more consistently after 6 years (23% decrease, *P* = 0.044, Figure 4B). Dh did not show any significant Stand or Period effect, but we found a significant Stand × Period interaction (Table 1 and Figure 3). *Post-hoc* tests revealed that in the drained stand, Dh was significantly larger before 2003 (68.29 μm ± 0.40) than for the period 2003–2010 (65.44 μm ± 1.63; *P* = 0.034). Welch’s tests did not show any significant change after the 2011 event in any of the stands (Figure 4C). Ks also showed a significant Stand × Period interaction only (Table 1 and Figure 3). In this case, a significant increase took place in the rewetted stand after 2003, so Ks values were larger for the period 2003–2010 (0.024 m^2^ s^–1^ MPa^–1^ ± 0.002) than before 2003 (0.021 m^2^ s^–1^ MPa^–1^ ± 0.002; *post-hoc* test, *P* = 0.015). CWT was the only parameter for which cDBH had a significant effect (Table 1 and Figure 3). We did not find any significant effect for Stand, Period, or their interaction on this parameter. The clear differences in raw CWT values between the drained and the rewetted stand (Figure 3) are driven by differences in DBH between the selected trees (the Stand effect disappeared from the models when accounting for cDBH, Supplementary Figure 12).

### Anatomical Trait and Density Relationships

**FIGURE 5:**
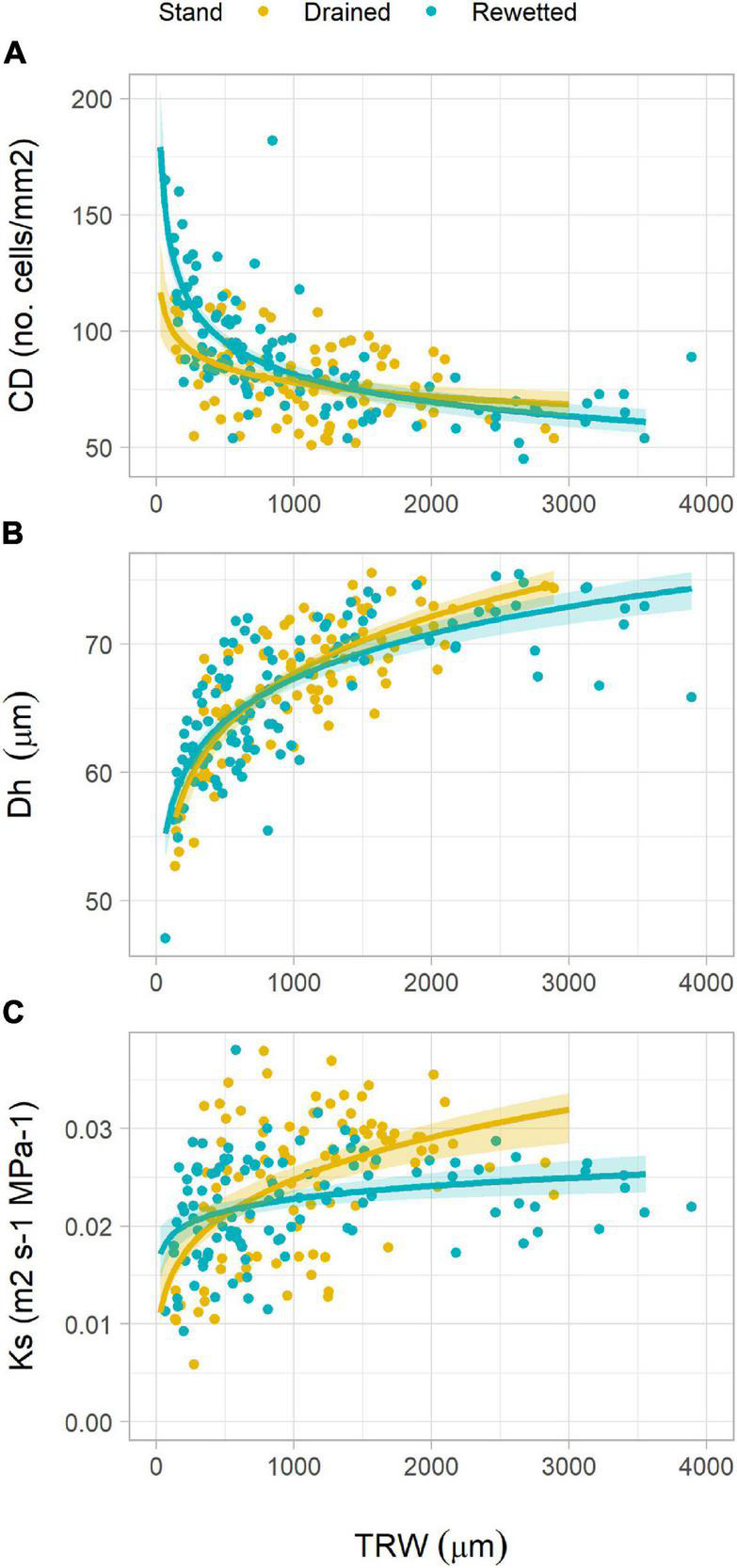
Relationship between tree-ring width (TRW) and **(A)** vessel density (CD), **(B)** hydraulically weighted mean vessel diameter (Dh), and **(C)** xylem-specific hydraulic conductivity at ring level (Ks) in both stands. Lines show regression fits and shadows indicate the 95% confidence interval of the model fit (refer to Table 2 for regression equations).

**FIGURE 6:**
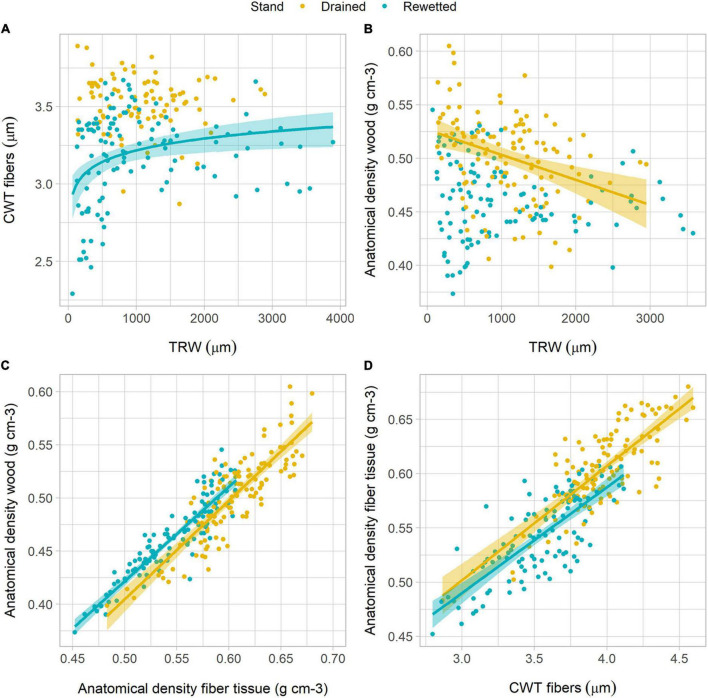
Relationship between anatomical density parameters, cell wall thickness (CWT), and tree-ring width (TRW) in both stands. **(A)** Relationship between CWT and TRW; **(B)** relationship between anatomical wood density and TRW; **(C)** relationship between anatomical wood density and the density of the fibers; **(D)** relationship between the density of the fibers and CWT. Lines show regression fits and shadows indicate the 95% confidence interval of the model fit (refer to Table 2 for regression equations).

The analysis of the relationship between anatomical traits showed some differences between the stands. CD decreased with increasing TRW following a power function in both stands. With lower TRW values, CD was higher in the rewetted stand than in the drained stand (Figure 5A and Table 2). Oppositely, Dh increased with TRW in a similar way in both stands (Figure 5B and Table 2), also following a power function. Ks increased with TRW following a power function in both stands, but for larger rings, the values were higher in the drained than in the rewetted stand (Figure 5C). CWT did not show a significant relationship with TRW in the drained stand (Table 2 and Figure 6A), but it increased with larger TRW in the rewetted stand (although values were quite variable, especially with smaller TRW). Wood anatomical density linearly decreased with increasing TRW in the drained stand and showed a power relationship with TRW in the rewetted stand, although values were especially variable with small TRW (Table 2 and Figure 6B). The anatomical wood density increased with the density of the fiber tissue in a similar way in both stands (same slope) but wood anatomical density values were larger in the rewetted than in the drained stand for the same fiber density (Table 2 and Figure 6C). The fiber density increased with CWT but, in this case, the fiber density values in the drained stand were larger than in the rewetted stand for the same CWT. Trees in the drained stand showed higher CWT values (Table 2 and Figures 3, 6D).

**TABLE 2:** Regressions among xylem anatomical traits, tree-ring widths (TRW), and anatomical density parameters.

Stand	Equation	*R* ^2^	*P*-value	Figure in the article
Drained	ln(CD) = 5.13-0.11 × ln(TRW)	0.13	<0.001	Figure 5A
Rewetted	ln(CD) = 5.93-0.22 × ln(TRW)	0.55	<0.001	Figure 5A
Drained	ln(Dh) = 3.56 + 0.09 × ln(TRW)	0.68	<0.001	Figure 5B
Rewetted	ln(Dh) = 3.69 + 0.08 × ln(TRW)	0.60	<0.001	Figure 5B
Drained	ln(Ks) = -5.85 + 0.31 × ln(TRW)	0.32	<0.001	Figure 5C
Rewetted	ln(Ks) = -4.51 + 0.10 × ln(TRW)	0.13	<0.001	Figure 5C
Drained	–	–	–	Figure 6A
Rewetted	ln(CWT) = 0.90 + 0.04 × ln(TRW)	0.12	<0.001	Figure 6A
Drained	Anatomical density = 0.53-2.34e-05 × TRW	0.11	<0.001	Figure 6B
Rewetted	–	–	–	Figure 6B
Drained	Anatomical density = -0.059 + 0.93 × fiber anatomical density	0.72	<0.001	Figure 6C
Rewetted	Anatomical density = -0.023 + 0.89 × fiber anatomical density	0.84	<0.001	Figure 6C
Drained	Fiber anatomical density = 0.186 + 0.11 × CWT	0.62	<0.001	Figure 6D
Rewetted	Fiber anatomical density = 0.198 + 0.10 × CWT	0.59	<0.001	Figure 6D

*TRW, tree-ring width; CD, vessel density; Dh, hydraulically weighted mean vessel diameter; Ks, theoretical xylem-specific hydraulic conductivity; CWT, cell wall thickness. Non-significant relationships are indicated with “−”. Power regression functions are transformed to log-log regressions for better clarity and interpretation of the models.*

Wood anatomical density profiles showed that fiber density variability strongly correlated with wood anatomical density (*r* = 0.87, *P* < 0.001 and *r* = 0.82, *P* < 0.001 in the drained and the rewetted stand, respectively) and was also its largest contributor (Supplementary Figure 13). Vessel density correlated with wood anatomical density to a lesser extent (*r* = 0.68, *P* < 0.001 and *r* = 0.44, *P* = 0.032 in the drained and the rewetted stand, respectively). Fiber density values were clearly higher in the drained than in the rewetted stand (*F*_1,8_ = 6.77, *P* = 0.032), but wood density and vessel density did not differ significantly between the stands (*F*_1,8_ = 2.59, *P* = 0.146, and *F*_1,8_ = 0.37, *P* = 0.560, respectively, Supplementary Figure 13).

## Discussion

### Growth Responses of Alder to Changing Water Regimes

Changing water tables are more important for alder growth than climate factors, such as precipitation or temperature *per se*. Previous dendroecological studies have found poor correlations between alder growth and climate variables, such as mean monthly temperature and monthly precipitation sum, whereas a relationship with hydrological changes has been widely reported (Laganis et al., 2008; Douda et al., 2009; Rodríguez-González et al., 2010, 2014). In fact, it is not surprising that site conditions overrule effects of climate in such azonal communities (Douda, 2008). In our study, correlations between growth and climate variables were weak at both stands (Supplementary Figure 7), while we found a clear growth decline in the rewetted stand after the extreme flooding in 2011 (but not in the drained stand, which received the same amount of precipitation; Figure 2).

Alder is a tree species well adapted to wet soil conditions, with enlarged stem lenticels, aerenchyma tissues in its roots, and the capacity to form adventitious roots after waterlogging (McVean, 1953; Gill, 1975). Yet, our results showed a growth decline in the rewetted stand after the extreme flooding in 2011 (Figures 2–4). Negative effects of waterlogging on alder growth have been previously reported in other studies (Rodríguez-González et al., 2010; Tulik et al., 2020) and the rewetting action that took place in 2003 at our study sites leading to a sudden and permanent flooding of an adjacent stand caused the death of all alder trees there (Bönsel, 2006). The negative effects of waterlogging, which are especially detrimental when occurring during the growing season (Kozlowski, 1997) as occurred in July 2011 in our study stands, are related to physiological dysfunctions caused by soil anaerobiosis, such as root hypoxia and growth inhibition (Kozlowski, 2002). In addition, wetter climate conditions and higher cloudiness may negatively affect alder photosynthesis and, in turn, oxygen transport to roots (Laganis et al., 2008). Low oxygen concentrations have also been reported to decrease nitrogenase activity in congeneric species, such as *Alnus maritima* (Marshall) Muhl. ex Nutt. (Kratsch and Graves, 2005) and *Alnus rubra* Bong (Batzli and Dawson, 1997). Flooding may reduce cambial activity even in flood-tolerant species due to anoxic conditions preventing active water transport needed for cell division and enlargement (Kozlowski, 1984; Peters et al., 2021) and may also reduce root growth in many woody plant species (Kozlowski, 1997). However, a study on root biomass of alder in the same study stands showed that tree fine root biomass in the rewetted stand was much greater than in the drained stand (Schwieger et al., 2021). Thus, the decreased aboveground growth in alder after 2011 may be more related to decreased photosynthetic rates (Graves et al., 2002) and root hydraulic conductance (Schmull and Thomas, 2000), a decrease in stem cambial activity (Kozlowski, 1984), or a compensation for the increase in biomass allocation to belowground organs rather than to reduced root growth. Interestingly, we found a growth increase in the drained stand after 2011 (Figure 2). The very wet year might have benefited trees at this stand since, as opposed to the rewetted stand, the drainage might have prevented an extreme flooding that year. Nevertheless, the positive response of tree growth in the drained stand might also be partly related to a competition release exerted by decaying ashes (*Fraxinus excelsior* L.). Ash dieback started in the area around 2002, caused by the fungus *Hymenoscyphus fraxineus*. The disease has been more pronounced in the last decade, and it is common to find fallen ash trees in the drained stand after a storm or a very windy episode.

### Xylem Anatomical Responses to Changing Water Regimes

Neither the growth decline after 2011 in the rewetted stand nor the growth increase in the drained stand were supported by a significant change in the diameter of the water-conducting vessels (Figure 3). Although vessel diameter showed a certain degree of interannual variability, it did not show a clear response to changing water tables (Figure 3). Only those anatomical traits more related to growth, such as the number of vessels (both absolute and per unit area) and the theoretical hydraulic conductivity (which is tightly related to the number of vessels, Supplementary Figure 9; Tyree and Zimmerman, 2002) responded to changing water regimes in a similar way as ring width. Tulik et al. (2020) studied vessel size adjustments to changing water table levels in alder trees of different health conditions and found a general decrease in vessel size under higher water table levels. Another study on flash-flood impacts on tree species anatomy found that the vessel size of alder decreased after wounding and identified alder as a particularly sensitive species to flash floods. Such a decrease in the vessel size, however, was linked to an injury and not to the changing water regime itself (Ballesteros et al., 2010). The lack of a decrease in vessel diameter coupled to the decreasing ring widths in the rewetted stand after the extreme flooding could indicate that, even though cambial activity was altered, it did not affect vessel development. The wood hydraulic architecture of alder was thus maintained under the changing water regimes. Xylem-specific hydraulic conductivity (Ks) was also remarkably independent of changing water table levels, and so was the vessel grouping (RVGI; Supplementary Figure 3), supporting the argument that hydraulic-related wood traits in alder are robust to changing water regimes. However, when exploring xylem-specific hydraulic conductivity in depth, and its relationships with ring width, we found different adjustments amongst stands. In favorable years (i.e., larger ring widths), trees at the drained stand showed larger Ks values than trees at the rewetted stand (Figure 5C). These differences were driven by slightly larger vessels and slightly more vessels with larger ring widths (neither significant when taken separately) at the drained stand compared to the rewetted stand (Figures 5A,B). This pattern, though, only occurred before the extreme flooding and disappeared after 2011 (Supplementary Figure 14), indicating that alder adjusted its hydraulic wood architecture to drier conditions before the flooding and to wetter conditions after it. Thus, despite unclear anatomical responses to changing water table levels, the hydraulic functioning of alder did show a plastic behavior tied to general growing conditions when facing such changes.

Even though microtopography was not found to be a clear predictor of alder growth in our rewetted stand (Wedell, 2020), the high variability in the anatomical data between trees in the same stand (Figure 3), together with the differences between data for the whole set of trees vs. the anatomical subset suggests that microsite conditions have a large influence on alder growth, wood density, and xylem anatomy. This is in accordance with the study by Gričar et al. (2013), who reported that anatomical characteristics and signals of hydrological changes in *Quercus robur* L. xylem rings showed a strong response to micro-environmental conditions. In addition, genetics might also play a role in explaining the high variability found (Pfenninger et al., 2021). We cannot exclude the possibility that such a high inter-individual variability might have obscured some patterns in alder anatomical responses to waterlogging.

### Alder Wood Density and Its Links to Anatomy

Wood density did not differ significantly between our forest stands, did not show strong correlations with climate, and did not show clear responses to the water regime. Although trees selected for the anatomical analysis showed consistently higher wood density values (both anatomical and radiographic density, Supplementary Figures 13, 15) and greater CWT (Figure 3) values in the drained than in the rewetted stand, these were likely driven by tree size, since anatomy-selected trees in the drained stand showed consistently larger DBH (Supplementary Figure 12). CWT has been shown to increase with DBH in clones of *Pinus tabuliformis* Carrière (Ouyang et al., 2018), and previous studies have reported relationships between tree size and wood density (Peters et al., 2020). However, when looking at the whole stand, these differences disappeared because tree size was more balanced (Supplementary Table 1 and Supplementary Figure 6).

The main variable explaining alder wood density fluctuations was CWT (Figures 6C,D). A previous study on *Fagus sylvatica* L. reported that interannual density variability was driven by a combination of vessel area and the number of vessels per unit area (Peters et al., 2020). In that study, a negative relationship between ring width and the number of vessels per unit area was found, which explained that wood density was greater in larger rings. Accordingly, our results show a negative relationship between the number of vessels per unit area and ring width (Figure 5A), but we only found a weak correlation between ring width and anatomical wood density in the drained stand, and this was negative (Figure 6B). While a negative wood density-ring width relationship has been reported for conifer species (Bouriaud et al., 2005; Franceschini et al., 2013; Pretzsch et al., 2018), this relationship is less clear in angiosperms, where no relationship (Bouriaud et al., 2004), negative relationships (Pretzsch et al., 2018), and positive relationships (Bergès et al., 2000; Peters et al., 2020) have been reported. In our study, wood density was mostly correlated with the density of the fibers in both stands which, in turn, was mostly explained by the thickness of the fiber cell walls (Figures 6C,D). A strong correlation between CWT and wood density has been well reported for conifers (Björklund et al., 2017, 2021), but remains less clear for angiosperms, where vessels, fibers, and parenchyma influence density in different ways. Interannual wood density variability in alder, although also influenced by the fiber to vessel area ratio (Supplementary Figure 16), is mostly driven by interannual variability in fiber CWT, which is more related to intrinsic physiological processes or biotic factors than climate variables or changing water regimes (Figure 3 and Supplementary Figure 12). This is also supported by the mild correlations found between climate variables (i.e., temperature and precipitation) and density, especially for MXD (Supplementary Figures 7, 8). Previous studies in the region have reported very weak correlations between climate variables (i.e., precipitation and temperature) or hydrological regimes and alder wood density (van der Maaten et al., 2015). It is noteworthy, though, that wood density responded in opposite ways to climate between stands to some extent. Thus, water regime would modulate the response of wood density in alder trees to climate, as reported by Elferts et al. (2011) for radial growth.

## Conclusion

Our study evidences the negative effects of sudden and permanent waterlogging for alder growth. Indeed, alder benefited from wet conditions, as indicated by increased growth in the drained stand after the extreme flooding, but only when these conditions did not lead to permanent waterlogging (as in the rewetted stand). The growth decline in the rewetted stand after the extreme flooding did not negatively affect the xylem anatomical architecture of alder, suggesting cell anatomical properties were not affected and species-specific thresholds in tolerance to waterlogging and soil anaerobiosis were not reached. Hydraulic-related xylem anatomical traits in alder were generally robust to water table changes, but hydraulic conductivity showed adjustments to changing water regimes when growing conditions were favorable. Altogether, our results provide evidence to the importance of monitoring rewetting actions to establish and maintain suitable water table levels for alder growth and functioning to restore alder peatland forests. Our results suggest that, with carefully planned rewetting actions, acclimation of existing alder stands to increasing water levels is possible without stand-replacing disturbance, although growth is still more optimal under drier conditions. Our study also provides a detailed understanding of the anatomical basis of alder wood density and the relationship between traits, showing a clear effect of CWT on alder wood density, as previously reported for conifer species.

## Data Availability Statement

Data supporting this publication are stored at https://github.com/anadon-rosell/Anadon-Rosell_et_al_2021_FPS.

## Author Contributions

AA-R, TS, and MW conceived and designed the study with input from GA and RP. AA-R, TS, and MS carried out the fieldwork. AA-R prepared and analyzed the samples, performed the statistical analyses, and wrote the manuscript with substantial advice from GA, RP, and TS. All authors contributed to the manuscript revision.

## Conflict of Interest

The authors declare that the research was conducted in the absence of any commercial or financial relationships that could be construed as a potential conflict of interest. The handling editor declared a past co-authorship with one of the authors GA.

## Publisher’s Note

All claims expressed in this article are solely those of the authors and do not necessarily represent those of their affiliated organizations, or those of the publisher, the editors and the reviewers. Any product that may be evaluated in this article, or claim that may be made by its manufacturer, is not guaranteed or endorsed by the publisher.
