# Supplementary material for: Growth and Wood Trait Relationships of Alnus glutinosa in Peatland Forest Stands With Contrasting Water Regimes
Source: Front Plant Sci. 2022 Jan 12;12:788106. doi: 10.3389/fpls.2021.788106 (PMC8790179; doi:10.3389/fpls.2021.788106)
Supplement: Supplementary file 1 [file Data_Sheet_1.PDF]

# *Growth and wood trait relationships of* Alnus glutinosa *in peatland forest stands with contrasting water regimes*

## *Supplementary material*

**Table S1.** Data on the study trees. In grey, anatomy-selected trees.

| Stand | TreeID | DBH (cm) | Height (m) |
|---|---|---|---|
| Drained | ADAG01 | 38.4 | 23.4 |
| Drained | ADAG02 | 35.5 | 19.1 |
| Drained | ADAG03 | 42 | 24.9 |
| Drained | ADAG04 | 41.3 | 25.4 |
| Drained | ADAG06 | 36.5 | 20.4 |
| Drained | 1643 | 42.5 | 27 |
| Drained | 1652 | 35.5 | 25.7 |
| Drained | 1680 | 55 | 28 |
| Drained | 1681 | 24 | 22 |
| Drained | 1683 | 29.5 | 24 |
| Drained | 1684 | 36 | 25 |
| Drained | 1688 | 48.5 | 26.8 |
| Drained | 1689 | 45.5 | 26.2 |
| Drained | 1695 | 40 | 24.6 |
| Drained | 1699 | 44.5 | 25.6 |
| Drained | ON1 | 35 | 26.5 |
| Rewetted | AWAG01 | 32.5 | 24.2 |
| Rewetted | AWAG02 | 23.1 | 19.1 |
| Rewetted | AWAG03 | 28.4 | 19.6 |
| Rewetted | AWAG04 | 35.5 | 24.4 |
| Rewetted | AWAG05 | 31.5 | 20.2 |
| Rewetted | 1840 | 34.5 | 20.6 |
| Rewetted | 1842 | 39 | 21.2 |
| Rewetted | 1843 | 28 | 23.8 |
| Rewetted | 1845 | 33 | 22.9 |
| Rewetted | 1846 | 30.5 | 23.1 |
| Rewetted | 1848 | 28.5 | 23.7 |
| Rewetted | 1849 | 34 | 22.4 |
| Rewetted | 1850 | 36.5 | 27 |
| Rewetted | 1855 | 40.5 | 27.5 |
| Rewetted | 1859 | 31 | 22.6 |
| Rewetted | 1864 | 30.5 | 21.4 |
| Rewetted | 1865 | 42 | 28.1 |
| Rewetted | 1869 | 35 | 24.4 |
| Rewetted | 1878 | 30 | 14.3 |
| Rewetted | 1880 | 29 | 29.1 |
| Rewetted | 1889 | 48.5 | 27.3 |
| Rewetted | 1892 | 34.5 | 22.9 |

**Table S2.** Results of the ANOVA tests for extra anatomical variables. cDBH was only kept in the models when it was significant or marginally significant ($P < 0.10$).

| Variable | cDBH | | | Stand | | | Period | | | Stand x Period | | |
|---|---|---|---|---|---|---|---|---|---|---|---|---|
| | df | F | P | df | F | P | df | F | P | df | F | P |
| CNo | 1,195 | 2.87 | 0.092 | 1,7 | 1.84 | 0.217 | 2,195 | 0.94 | 0.392 | 2,195 | 0.52 | 0.594 |
| Kh | - | - | - | 1,8 | 0.24 | 0.639 | 2,221 | 1.26 | 0.286 | 2,221 | 1.43 | 0.243 |
| RVGI | - | - | - | 1,8 | 1.19 | 0.308 | 2,221 | 0.02 | 0.977 | 2,221 | 0.38 | 0.684 |

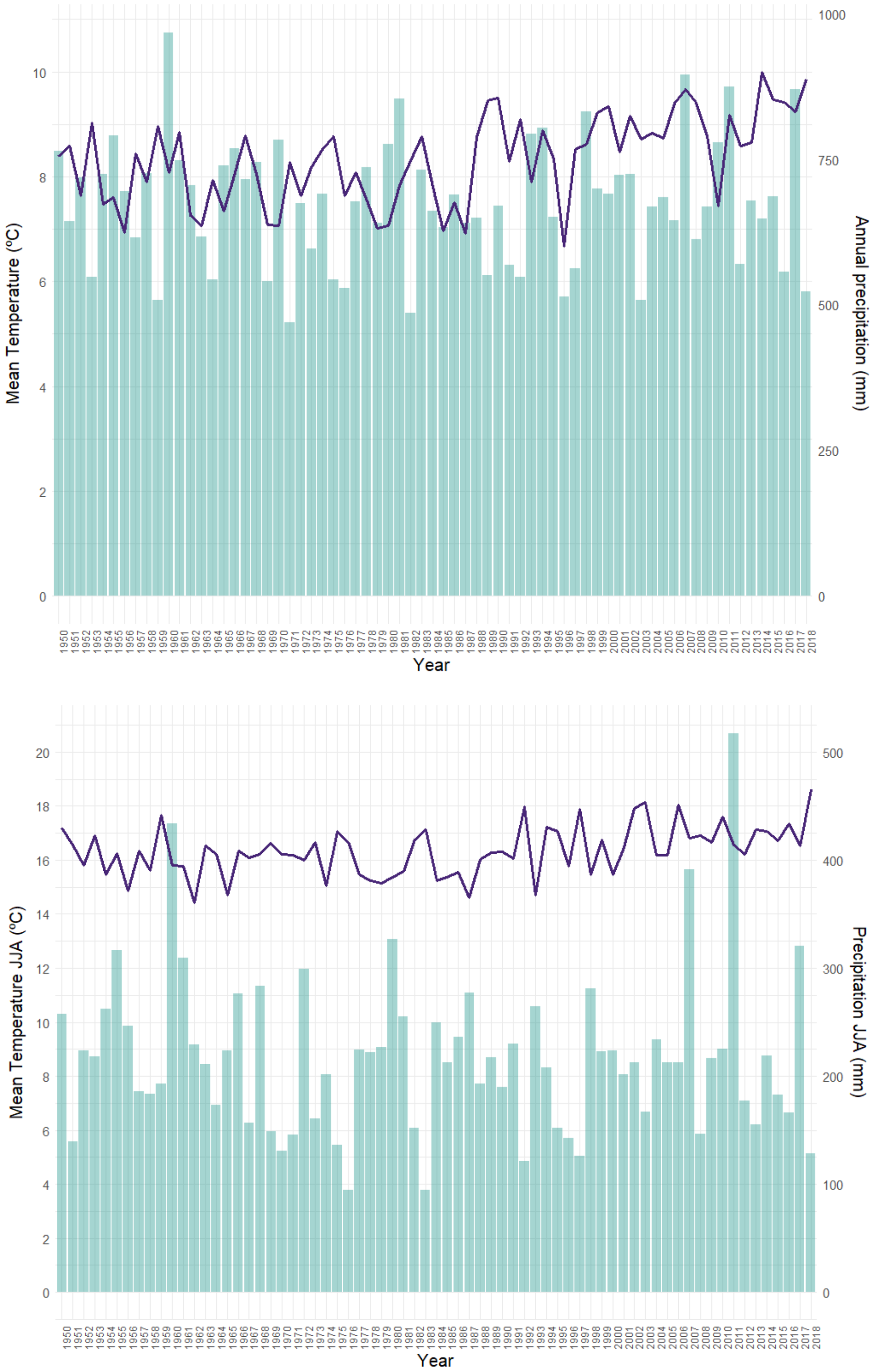


**Figure S1.** Climate data for the study site over the period 1950-2018. On top, mean annual temperature and total annual precipitation, at the bottom mean temperature and precipitation sums for the summer months (JJA). Purple lines show temperature data; green bars show precipitation data.

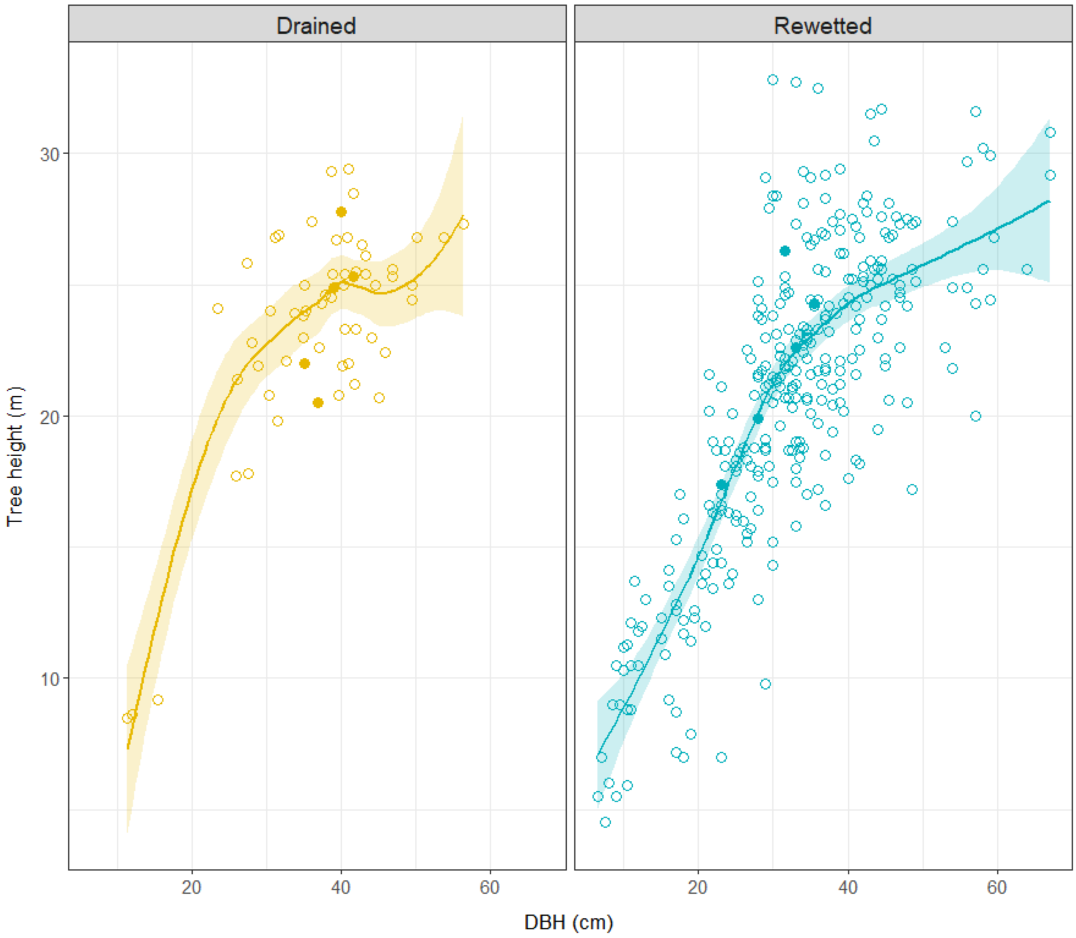


**Figure S2.** Relationship between tree height and tree DBH for all trees present at each study stand (thus, this figure includes more trees than those measured in this study). Filled circles correspond to the anatomy-selected trees.

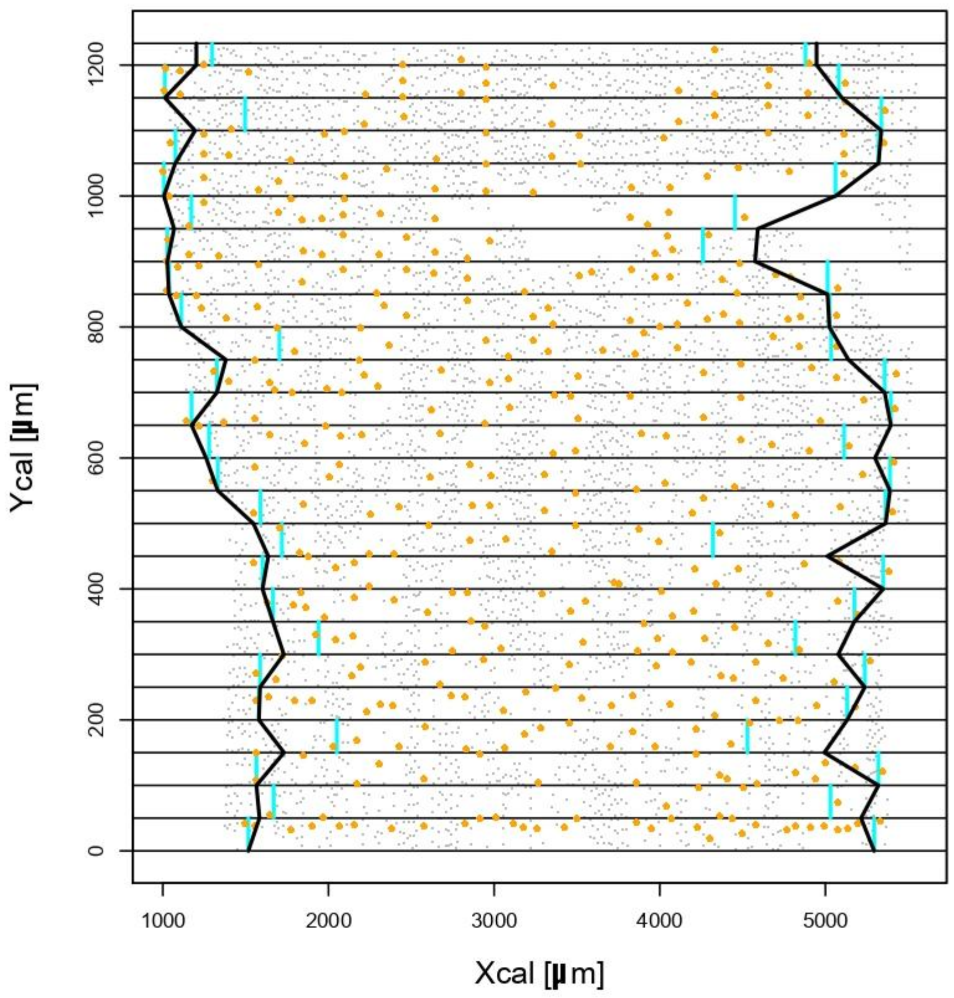


**Figure S3.** Representation of a tree ring divided into 50 µm-wide sectors parallel to the ring border for the construction of wood density profiles (this image shows ring built in 1999 from tree ADAG04). Big orange dots represent vessels and small grey dots represent fibers. The size of vessels and fibers is not depicted in this representation, only their centroid position within the ring.

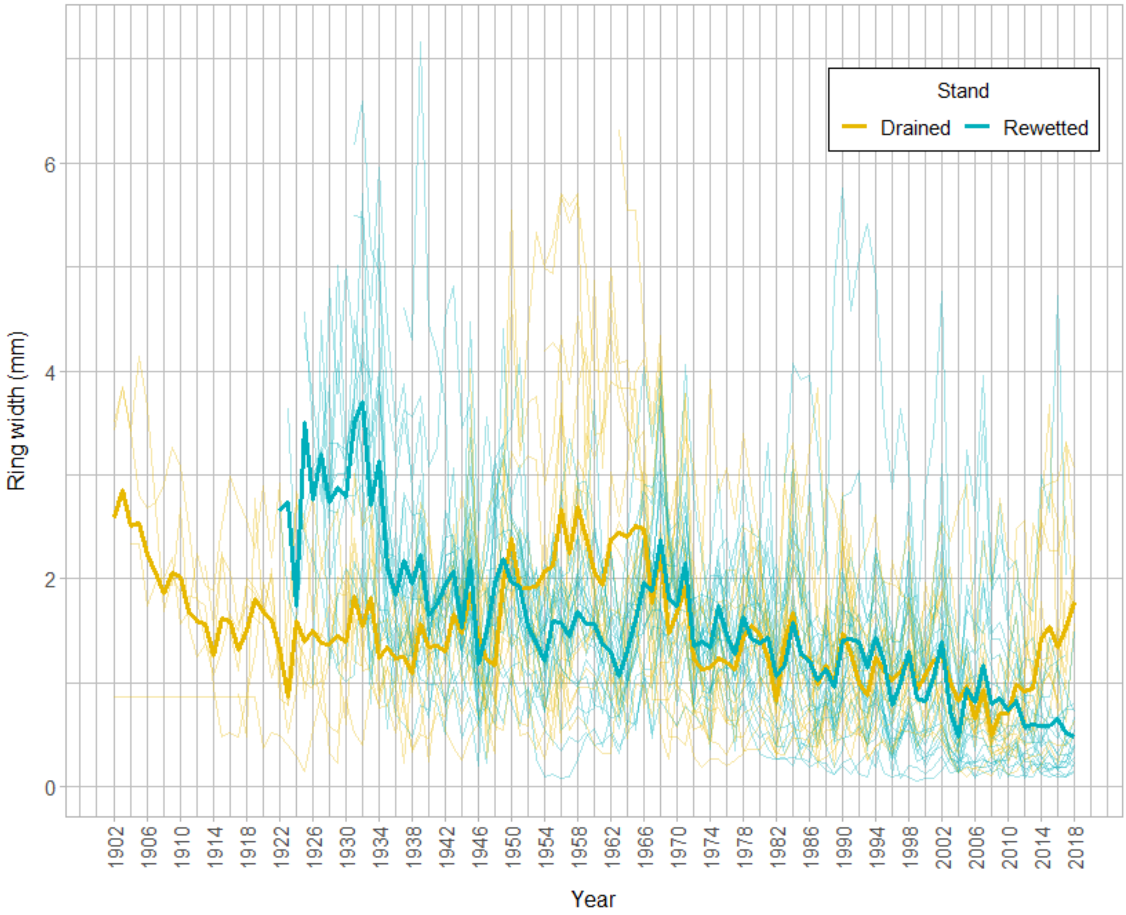


**Figure S4.** Alder tree-ring width (TRW) chronology for both stands. Individual tree chronologies are shown in thin lines and mean average chronologies for each stand are shown in thick lines.

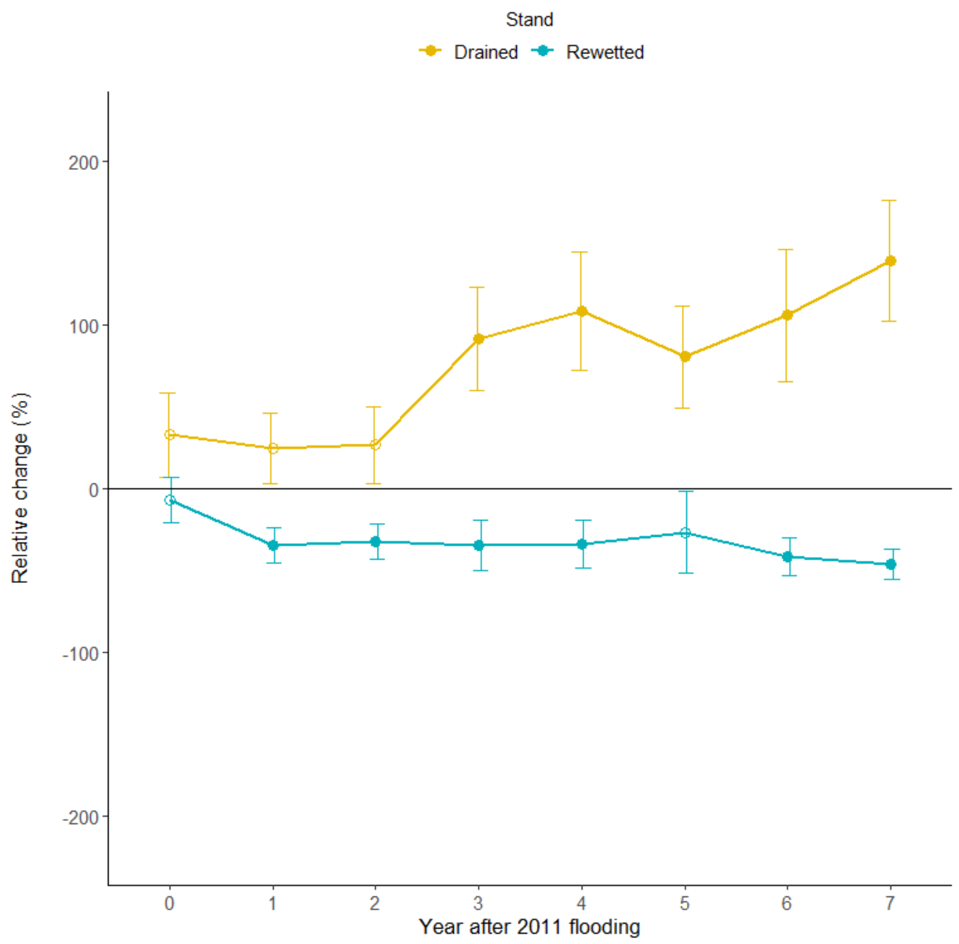


**Figure S5.** Relative change in TRW after 2011 flooding respect to the reference (mean of six years prior to 2011, 0.74 ± 0.07 mm in the drained stand, 0.88 ± 0.06 mm in the rewetted) for trees at stand level. Year 0 corresponds to 2011. Empty circles represent non-significant change values ($P \geq 0.05$), filled circles represent significant changes ($P < 0.05$).

**a)**

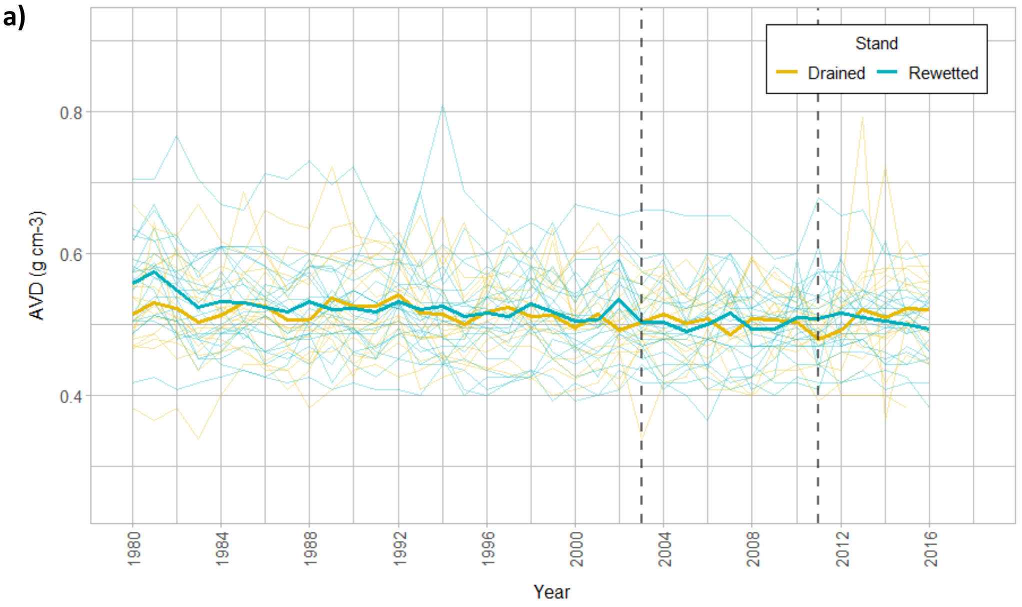


**b)**

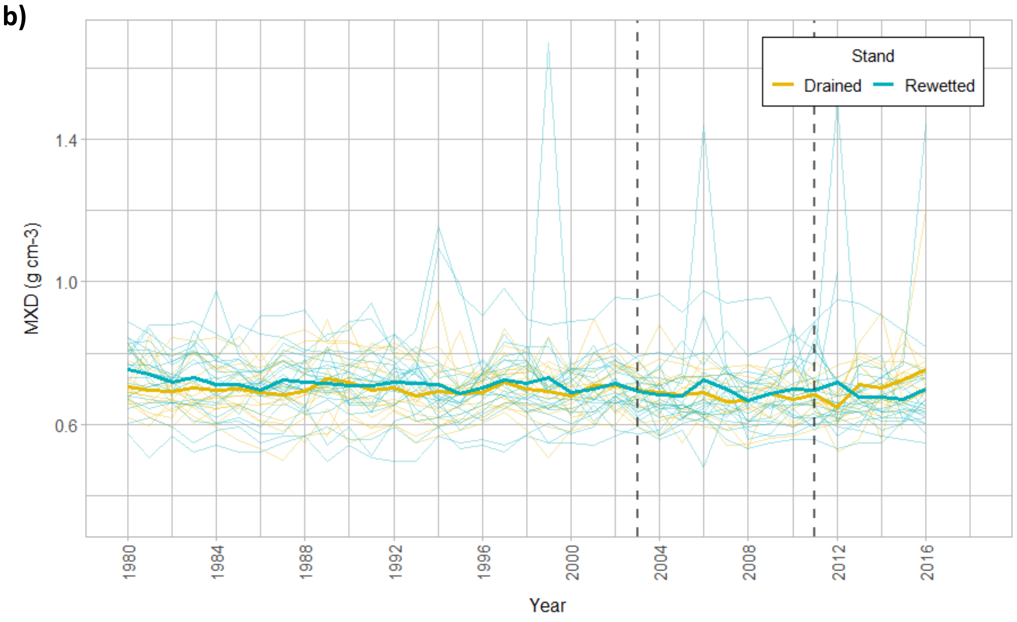


**Figure S6.** (a) Average ring density (AVD, g cm$^{-3}$) and (b) maximum latewood density (MXD, g cm$^{-3}$) measured in alder x-radiographs. Individual tree chronologies are shown in thin lines and mean average chronologies for each stand are shown in thick lines.

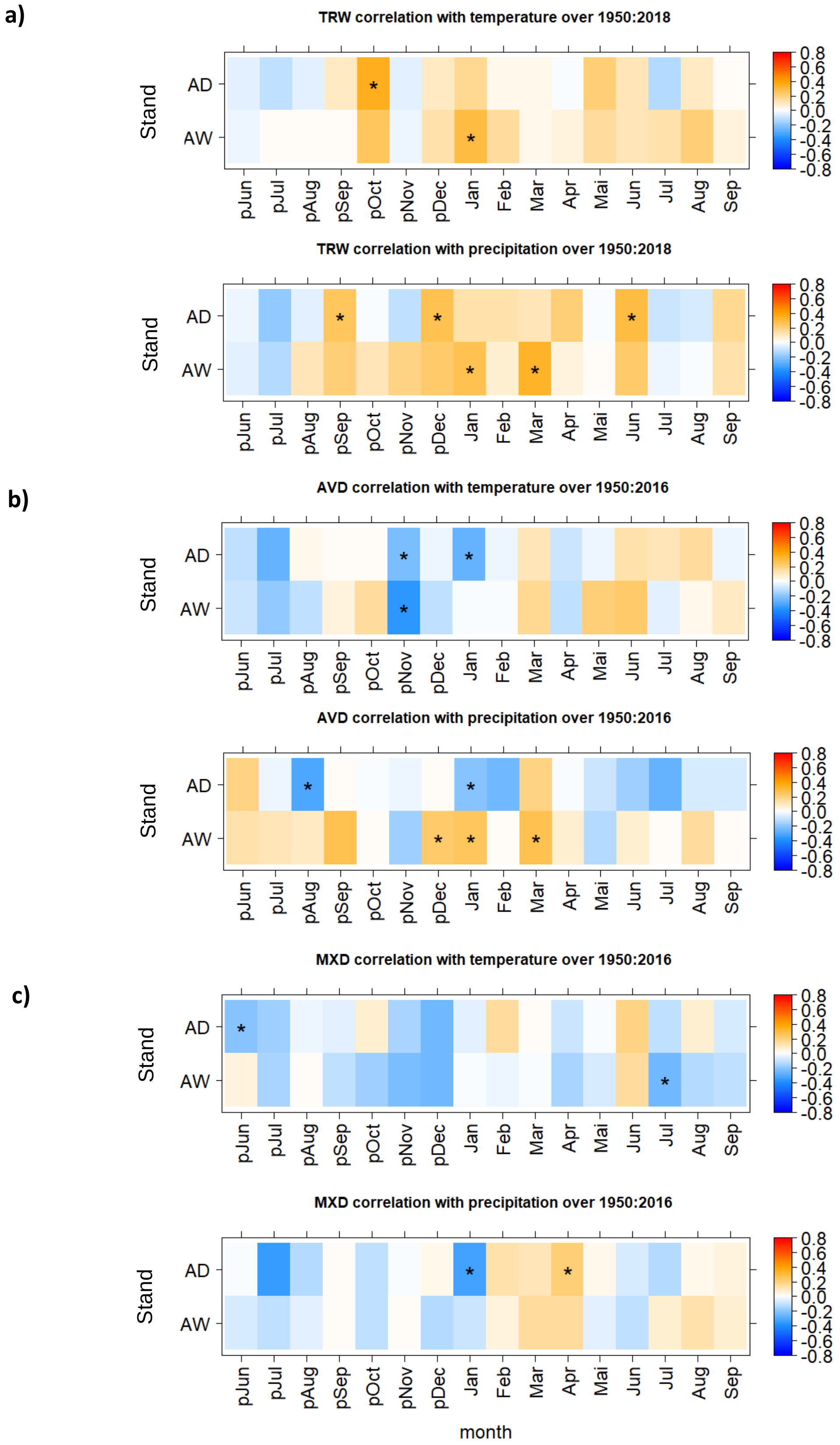


**Figure S7.** Monthly climate-growth correlations for the period 1950-2018 (a) and climate-density correlations for the period 1950-2016 (b, AVD = average ring density; c, MXD = maximum latewood density) in both stands (AD = drained, AW = rewetted). Asterisks indicate significant correlations ($P < 0.05$).

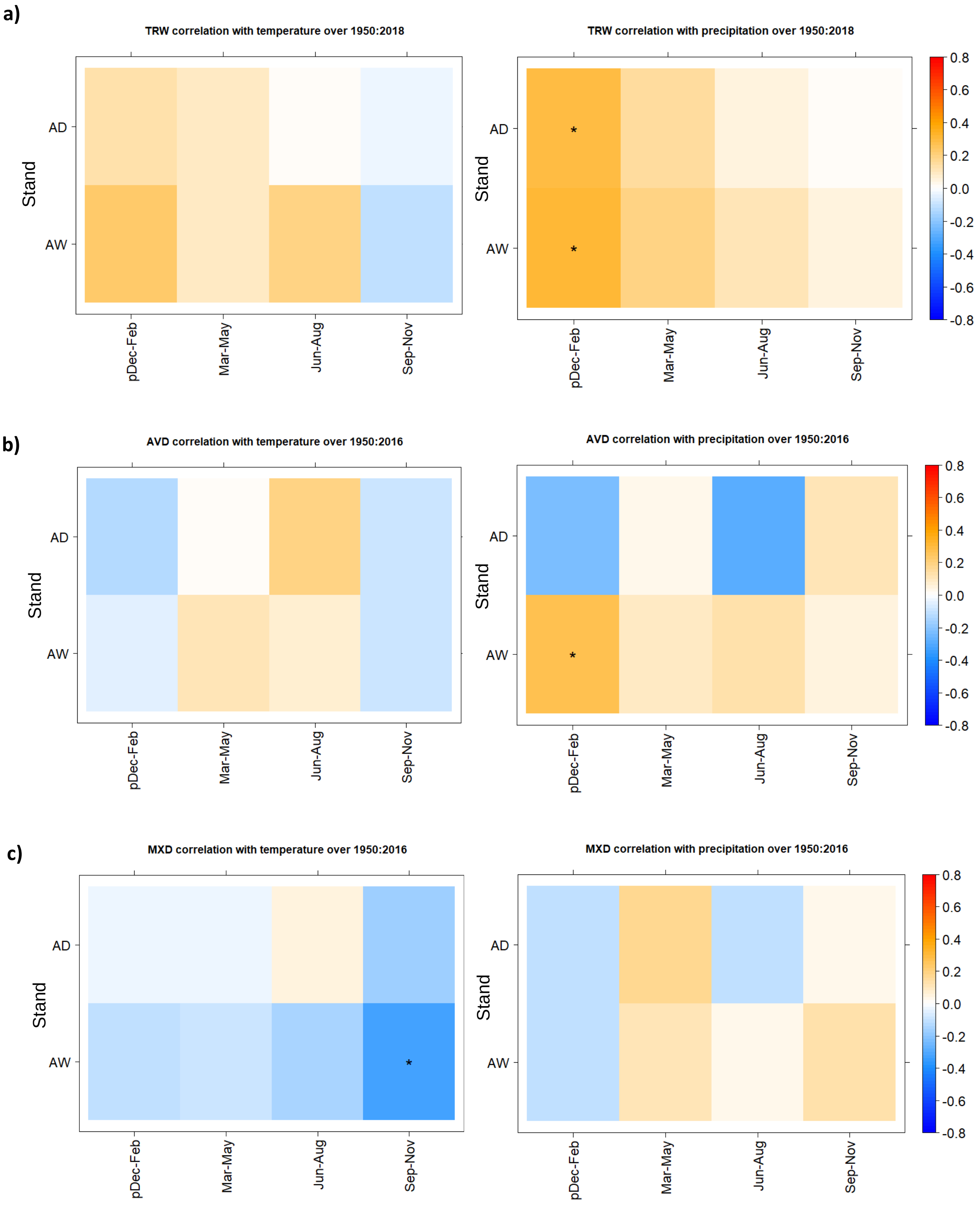


**Figure S8.** Seasonal climate-growth (a) and climate-density (b, AVD = average ring density; c, MXD = maximum latewood density) correlations for the period 1950-2018 (only until 2016 for the density parameters) in both stands (AD = drained, AW = rewetted). Asterisks indicate significant correlations ($P < 0.05$).

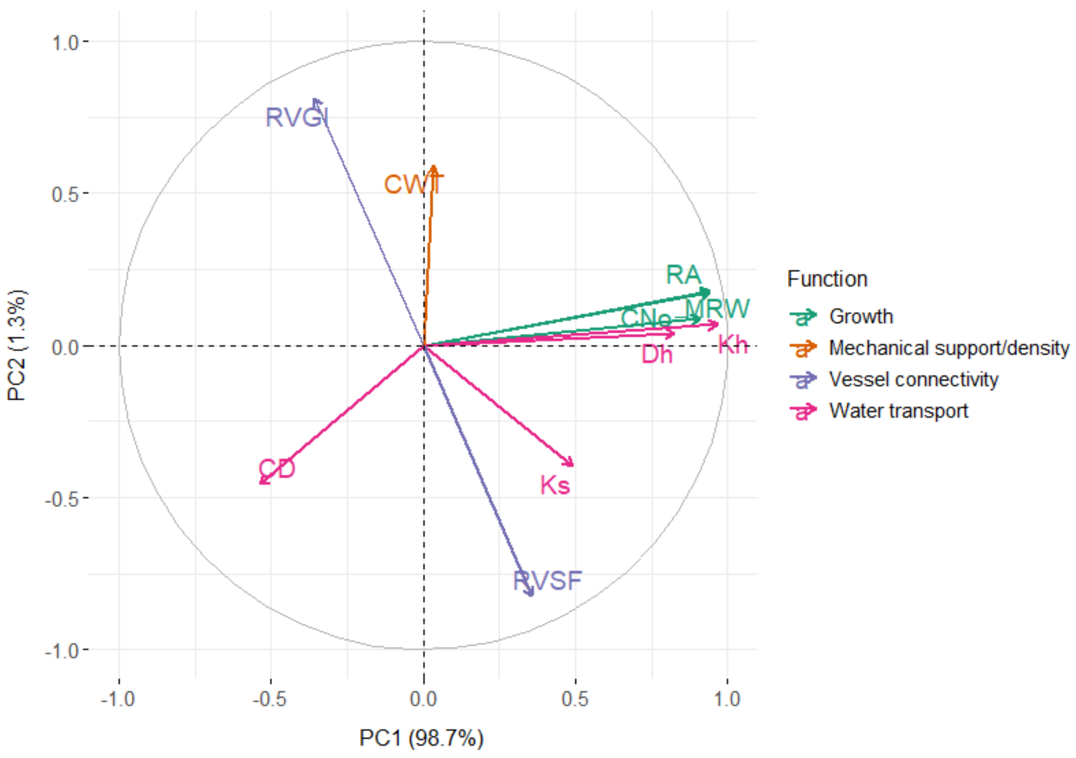


**Figure S9.** PCA for all the anatomical and growth traits.

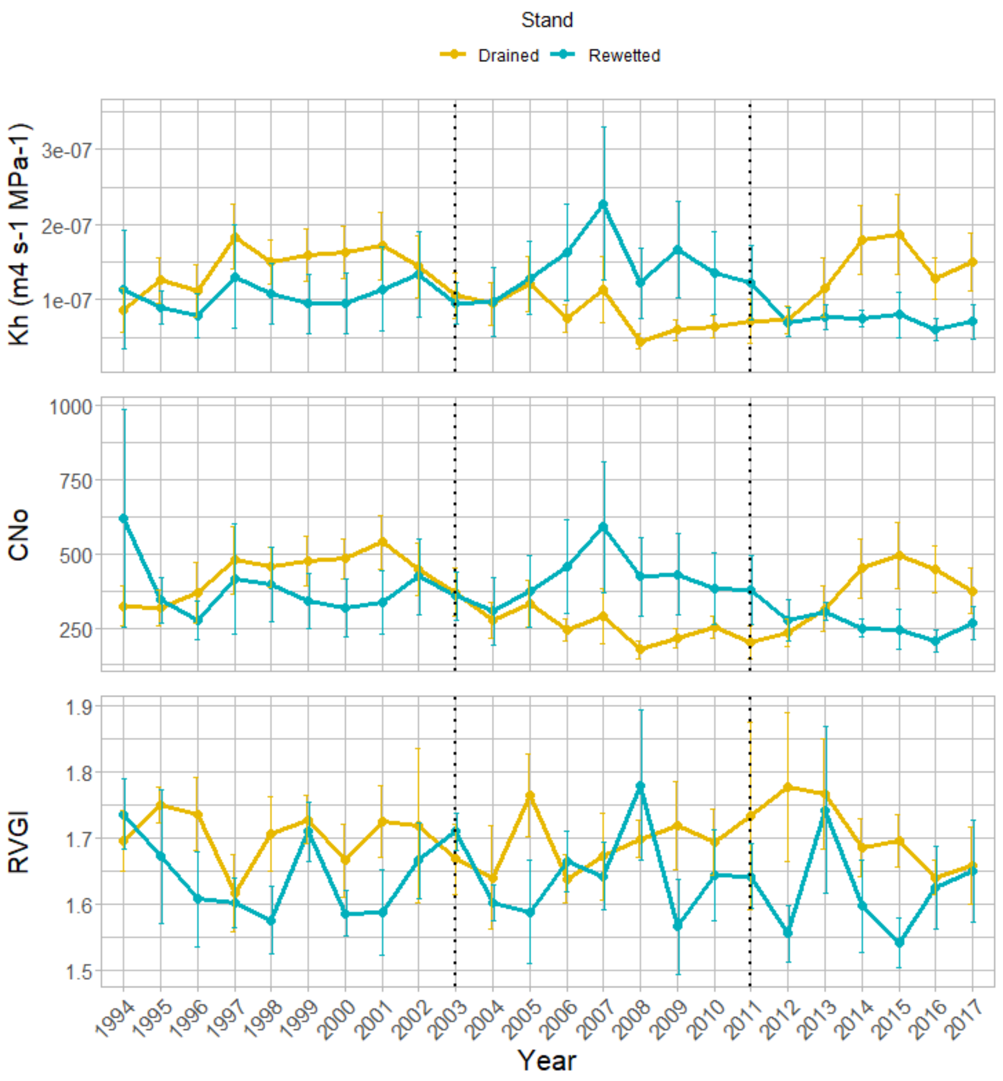


**Figure S10.** Theoretical hydraulic conductivity (Kh), vessel number (CNo) and vessel grouping index (RVGI) at the study stands (mean ± SE). Vertical dotted lines indicate the year of rewetting (2003) and the extreme flooding year (2011). See Table S2 for statistical differences.

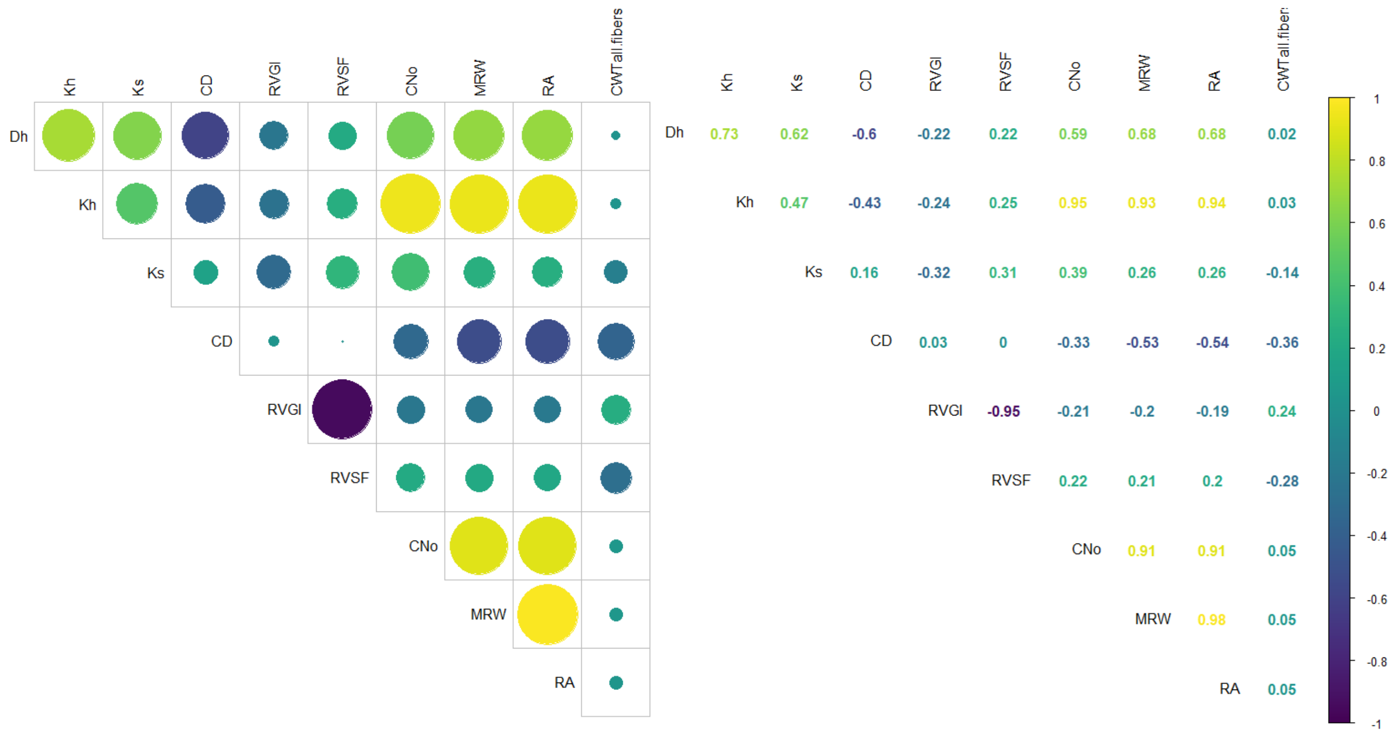


**Figure S11.** Spearman correlations (r) between xylem anatomical traits.

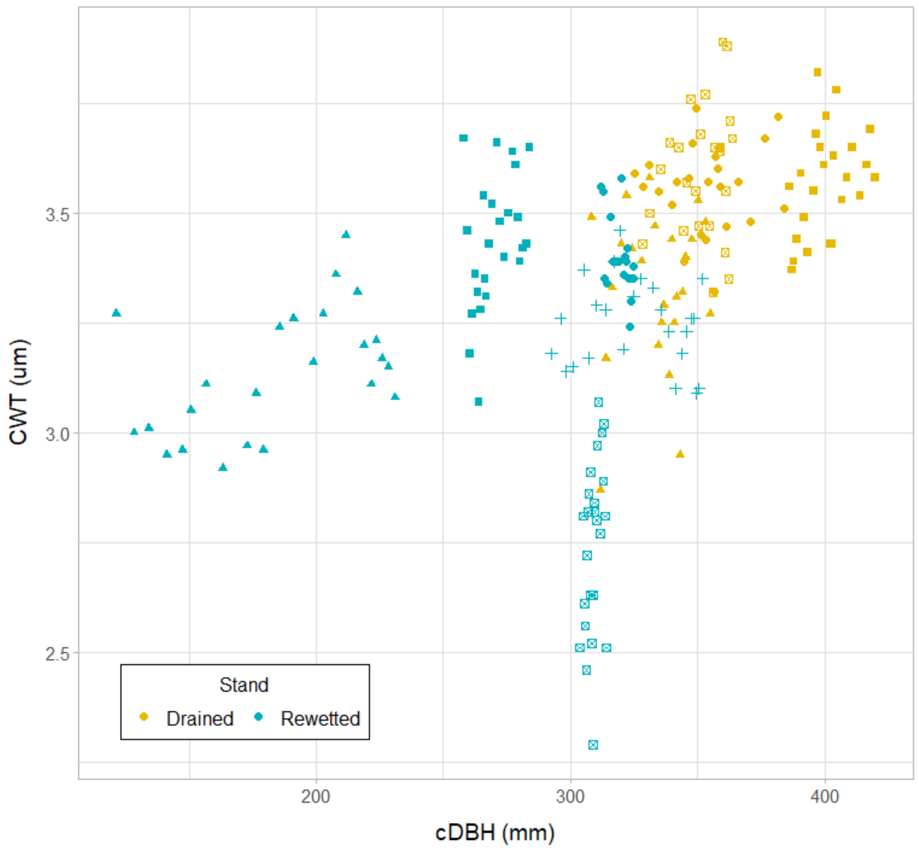


**Figure S12.** Relationship between cell wall thickness (CWT) and cumulative DBH (cDBH) at each stand. Each symbol indicates an individual tree.

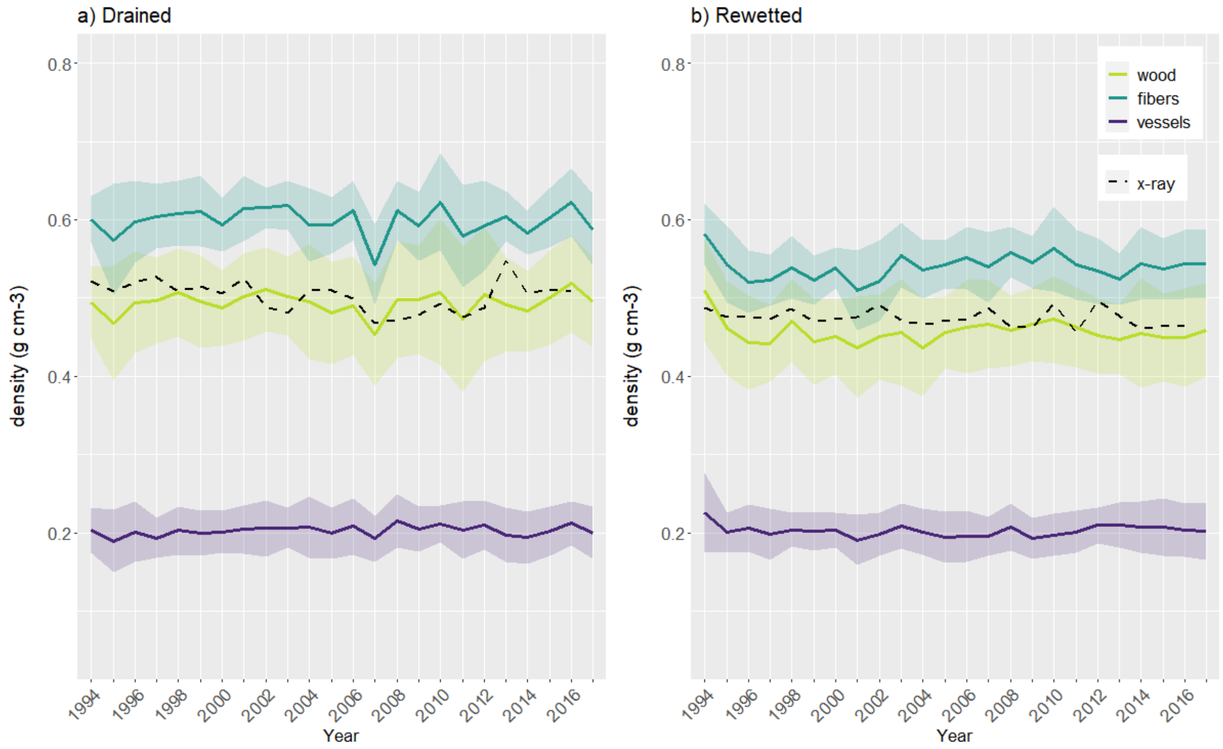


**Figure S13**. Anatomical wood density (means ± SD) in the (a) drained and in the (b) rewetted stand fractionated for wood (i.e., total anatomical density), fibers and vessels. The black dotted line shows the x-ray density. See the main text for correlation coefficients between parameters.

Wood anatomical density was close to x-ray density in absolute terms for both stands (mean ± SD in the drained stand were 0.49 ± 0.01 g cm-3 and 0.49 ± 0.01 g cm-3, respectively; and in the rewetted stand 0.46 ± 0.02 g cm-3 and 0.47 ± 0.01 g cm-3, respectively) but they were not significantly correlated ($r$ = 0.19, $P$ = 0.38, and $r$ = 0.31, $P$ = 0.14 in the drained and the rewetted stand, respectively). The fact that x-ray images were taken from a separate core and that the variability in x-ray density values was much higher than that of wood anatomical density makes it difficult to use the comparison between x-ray and wood anatomical density.

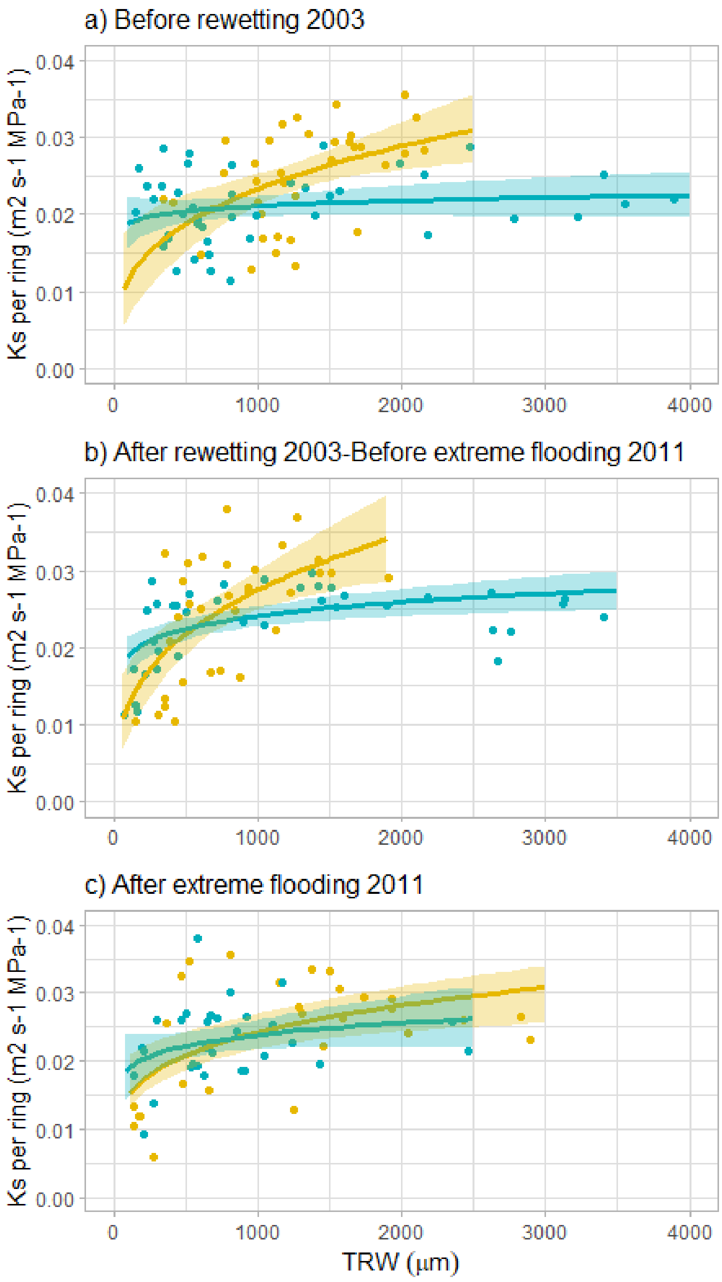


**Figure S14.** Relationship between Ks and TRW (a) before 2003, (b) after 2003, before the extreme year 2011, (c) after the extreme flooding in 2011. Lines show regression fits and shadows indicate the 95% confidence interval. Colour orange corresponds to the drained stand, colour blue to the rewetted.

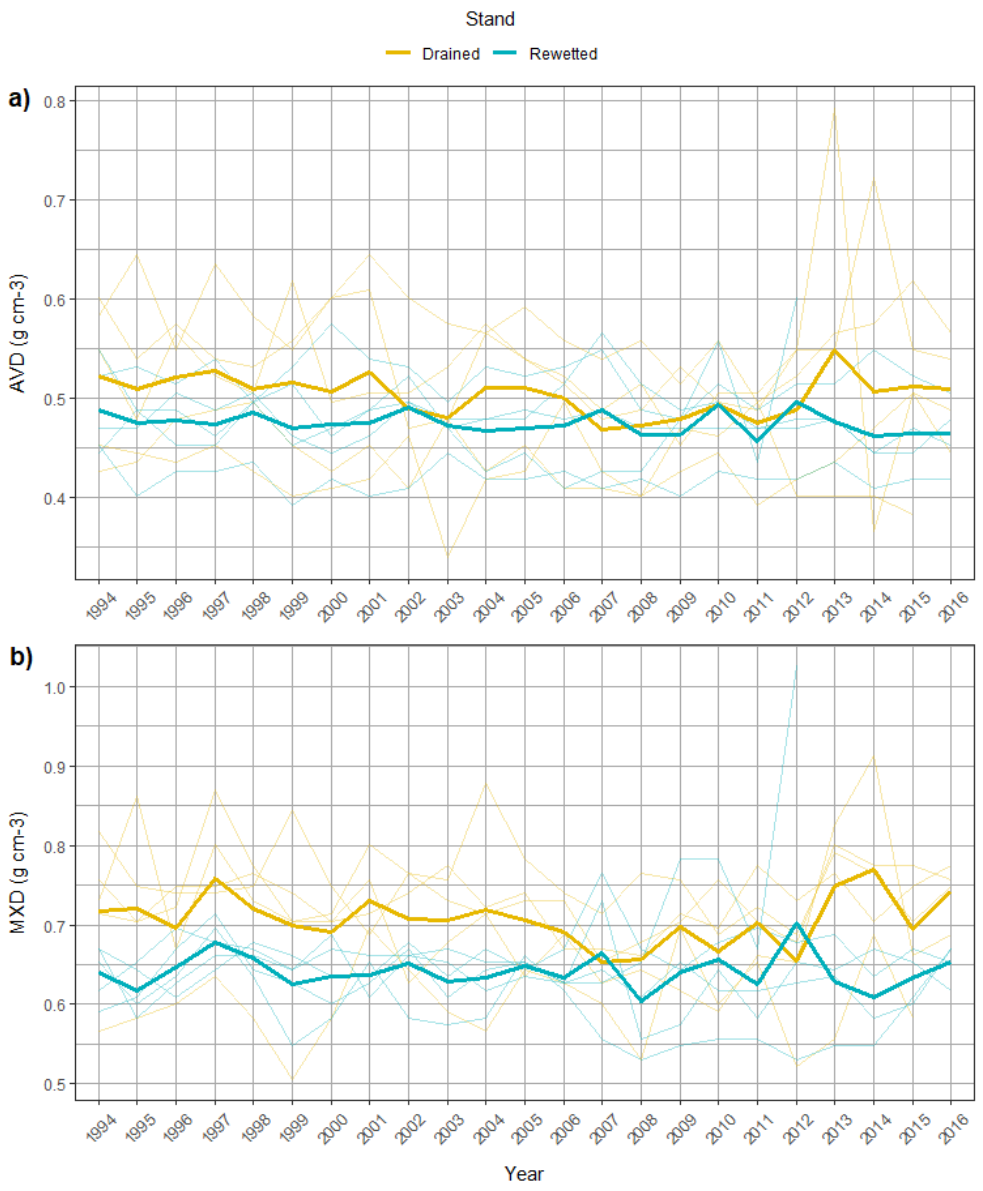


**Figure S15.** (a) Average ring density (AVD, g cm$^{-3}$) and (b) maximum latewood density (MXD, g cm$^{-3}$) measured in alder x-radiographs for the anatomy-selected trees. Individual tree chronologies are shown in thin lines and mean average chronologies for each stand are shown in thick lines.

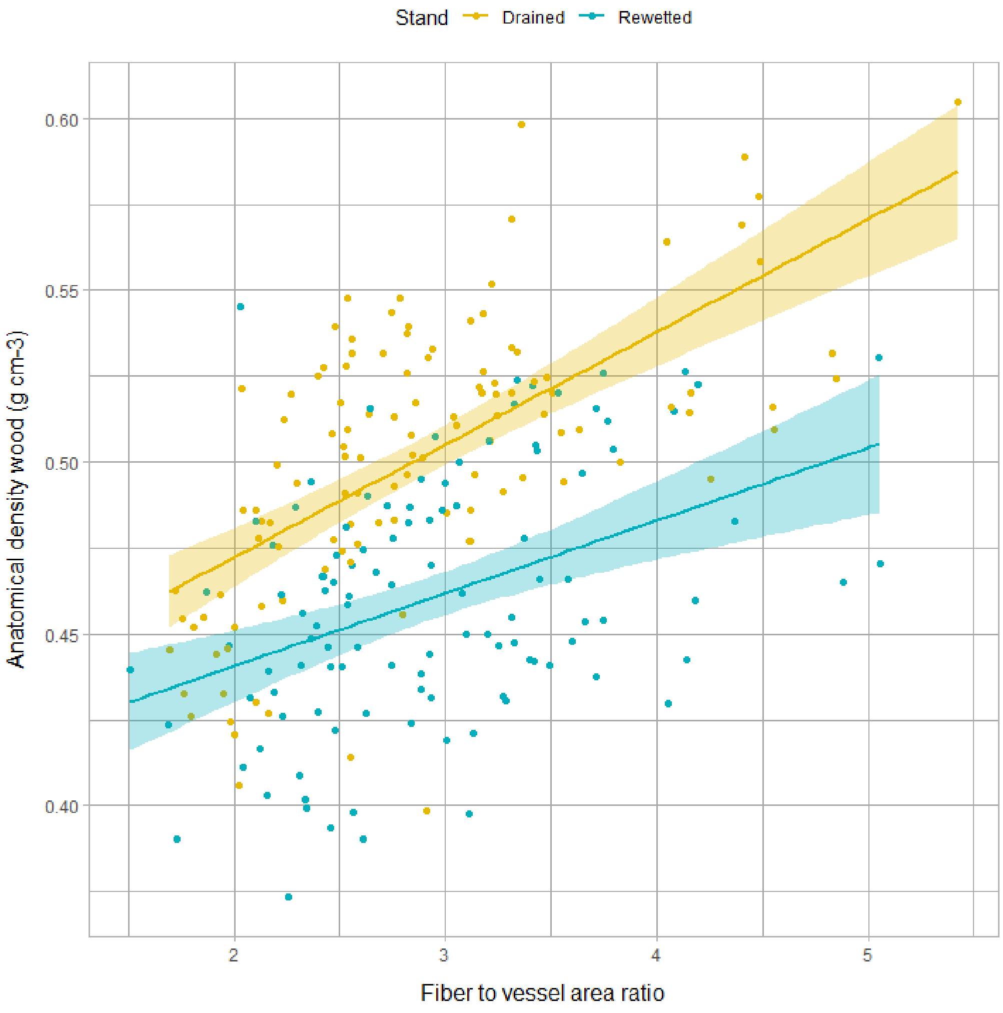


**Figure S16.** Relationship between anatomical wood density and the ratio between the area occupied by fibers and the area occupied by vessels in alder tree rings at each stand. Drained stand: $R^2$ = 0.40, P-value <0.001; Rewetted stand: $R^2$ = 0.16, P-value <0.001.
